# DnaA binding to *oriCII* regulates transcription from *Pseudomonas aeruginosa gidAB*-*parAB* locus linked to the chromosome segregation and streptomycin resistance

**DOI:** 10.1038/s41598-026-52016-6

**Published:** 2026-05-19

**Authors:** Pawel Wawrzyniak, Adam Kawalek, Aneta Szulc, Monika Glinkowska, Grazyna Jagura-Burdzy, Aneta Agnieszka Bartosik

**Affiliations:** 1 Institute of Biochemistry and Biophysics, Polish Academy of Sciences, Warsaw, Poland; 2 Department of Bacterial Genetics, Institute of Microbiology, Faculty of Biology, University of Warsaw, Warsaw, Poland; 3 Department of Bacterial Molecular Genetics, Faculty of Biology, University of Gdansk, Gdansk, Poland

**Keywords:** DnaA, Transcription factor, Streptomycin resistance, *Pseudomonas aeruginosa*, *oriC*, Chromosome segregation, Genetics, Microbiology, Molecular biology

## Abstract

In many bacteria, the DnaA protein, responsible for chromosome replication initiation, also acts as a transcription factor regulating its own gene expression and the expression of other loci. Here, we show that in the opportunistic human pathogen *Pseudomonas aeruginosa*, DnaA directly regulates the *gidAB-parAB* operon. DnaA binds to the DnaA boxes preceding the promoter located upstream of the *gidA* gene within *oriCII* and deletions in this region impair *gidAB-parAB* expression and reduce intracellular ParB levels. Additional regulatory elements, including three internal promoters, further contribute to the multilayered regulatory scheme of the *gidAB-parAB* operon. Since the products of *gidA* and *gidB* (glucose-inhibited cell division proteins) are important for cell growth, division, and survival, and those of *parA* and *parB* are central to chromosome segregation, regulation of this operon by DnaA suggests a functional link between replication initiation and the regulation of essential cell-cycle-related processes. These findings indicate a regulatory role for the *oriCII* region and a new function for DnaA in coordinating the gene expression required for proper replication, chromosome segregation, and bacterial growth in *P. aeruginosa*. Our data are also clinically significant, demonstrating that *P. aeruginosa gidB* gene disruption results in higher levels of streptomycin resistance.

**Supplementary Information:**

The online version contains supplementary material available at 10.1038/s41598-026-52016-6.

## Introduction

In the vast majority of bacteria, the DnaA protein orchestrates the initiation of chromosome replication^1^. DnaA interacts with DNA within the chromosome replication initiation region (*oriC*), and this binding is essential for DNA unwinding and the recruitment of replication machinery^2^. DnaA activity is modulated by its binding to ATP or ADP and oligomerization state^1^. DnaA-ATP and DnaA-ADP bind to DNA 9-mer sequences with consensus TTWTNCACA, known as DnaA boxes. High-affinity 9-mers are recognized by both DnaA-ATP and DnaA-ADP, but only DnaA-ATP can recognize the low-affinity DnaA boxes^3,4^. In addition, DnaA-ATP also binds single-stranded DNA sequences containing 6-mers with consensus AGATCT^5^. Finally, only the ATP-bound form of DnaA is competent for replication initiation^6,2^. DnaA activity is regulated not only by nucleotide switch but also by DnaA titration via DnaA boxes located outside the *oriC* region^7,8^. The *datA* locus in *Escherichia coli* contains multiple DnaA boxes, playing a role in sequestration of DnaA, and preventing premature initiation events^8,9^. In *Bacillus subtilis*, the *oriC*-proximal DnaA-box clusters also negatively regulate replication initiation as the deletion of six DnaA-box clusters was shown to cause premature replication initiation^10^.

DnaA is not only the main chromosome replication initiator, but also a transcription factor involved in regulating the expression of various genes by binding to DNA boxes located within promoter regions of target genes^11^. It can act either as a transcription repressor or as an activator^12^. It has also been proposed that DnaA may function as a transcription terminator^13^. DnaA negatively regulates its own expression^11^. In addition, it influences the expression of various genes involved in DNA replication, repair and cytokinesis^11^. The intracellular level of DnaA and nucleotide switch from DnaA-ATP to DnaA-ADP affects this transcription factor activity. For example, in *E. coli*, DnaA-ATP fully represses its own expression. In contrast, DnaA-ADP represses *dnaA* transcription at 40%^5^. Conversely, the *E. coli* ribonucleotide reductase *nrdAB* promoter is activated by DnaA-ADP (or low levels of DnaA-ATP)^14,15^ and is repressed by high levels of DnaA-ATP^15^. These examples illustrate how, in some bacteria, intracellular DnaA levels and nucleotide state fine-tune gene expression, suggesting an interconnection between DnaA-dependent gene expression and cell cycle related processes^11^.

In most bacteria, the *dnaA* gene is located within a cluster of highly conserved genes *rnpA*-*rpmH*-*dnaA*-*dnaN*-*recF*-*gyrB*, in proximity to the *oriC* sequence. In many bacteria, another region containing *gidAB* and *parAB* genes is also genetically linked to *oriC*^16,17^. The *gidAB* operon was first described in *E. coli* as a sequence responsible for glucose-mediated inhibition of cell division^18^. The *gidA* gene, initially characterized in *E. coli* as glucose inhibited division protein A (GidA) also known as *mnmG*, encodes a tRNA uridine 5-carboxymethylaminomethyl modification enzyme with FAD binding activity^19^. The deletion of *gidA* affects multiple phenotypes, including virulence^20^. The *gidB* (encoding glucose-inhibited cell division protein B, GidB), also referred to as *rsmG*, encodes a member of a large family of S-adenosylmethionine (SAM)-dependent methyltransferases functioning in ribosome function and translational accuracy. It exhibits a (SAM)-dependent 7-methylguanosine (m7G) methyltransferase activity responsible for methylation of 16 S rRNA at specific sites, thereby influencing translational fidelity and protein synthesis^21,22^ as well as antibiotic resistance^23,24^.

Concomitantly, protein products of *parA* and *parB* participate in early stages of chromosome segregation, as components of tripartite ParAB-*parS* systems^25^. These systems consist of two genes, *parA* and *parB*, organized in a single transcriptional unit, encoding ParA and ParB proteins, respectively. Multiple cognate *parS* DNA sequences are distributed around the chromosome mainly clustering in the *ori* domain^25^. ParB is a DNA-binding protein which recognizes and binds to the *parS* palindromic DNA sequence as a dimer. Cytidine triphosphate (CTP) binding causes ParB clamp formation, release from *parS*, and spreading on adjacent DNA^26^. Subsequent ParB multimerization causes the formation of complex 3D DNA-protein structures. ParB interacts with the motor protein ParA, a Walker-type ATPase, which drives ParB-DNA complex separation and consequently *ori* domain positioning at opposite cell poles^27^.

Interestingly, *P. aeruginosa* carries an additional replication origin-like region, termed *oriCII* spanning the 5’ part of the *gidA* ORF^28^. It was demonstrated that an 836 bp sequence fragment containing *oriCII*, when cloned in a plasmid vector, functions as a replication *origin*^28^. Nevertheless, only the *oriCI* is necessary and sufficient to initiate *P. aeruginosa* chromosome replication, whereas the function of *oriCII* remains unknown^28^. Both *oriCI* and *oriCII* contain 9 bp sequences resembling DnaA protein binding sites (DnaA-boxes) homologous to the consensus sequence TTWTNCACA^29,30^. The occurrence of *oriCI* and *oriCII* is not only characteristic of *P. aeruginosa*. This dual-origin configuration has recently been identified in 88 of 91 examined genomes of species of the Genome Taxonomy Database (GTDB)-family Pseudomonadaceae (including different *Pseudomonas* species) and in 138 of 163 examined genomes of a sister clade of the Halomonadaceae^31,32^.

In this study, we show that transcription of the *P. aeruginosa gidAB*-*parAB* operon is controlled by the DnaA protein, which directly interacts with the promoter region containing five DnaA boxes. We also demonstrate the presence of additional regulatory elements that contribute to *gidAB*-*parAB* operon control. We further show that disruption of the *P. aeruginosa gidAB*-*parAB* operon has clinically relevant implications, resulting in high level streptomycin resistance.

## Results

### Genetic organization of *P. aeruginosa oriC* region with *gidAB – parAB* genes

**Fig. 1:**
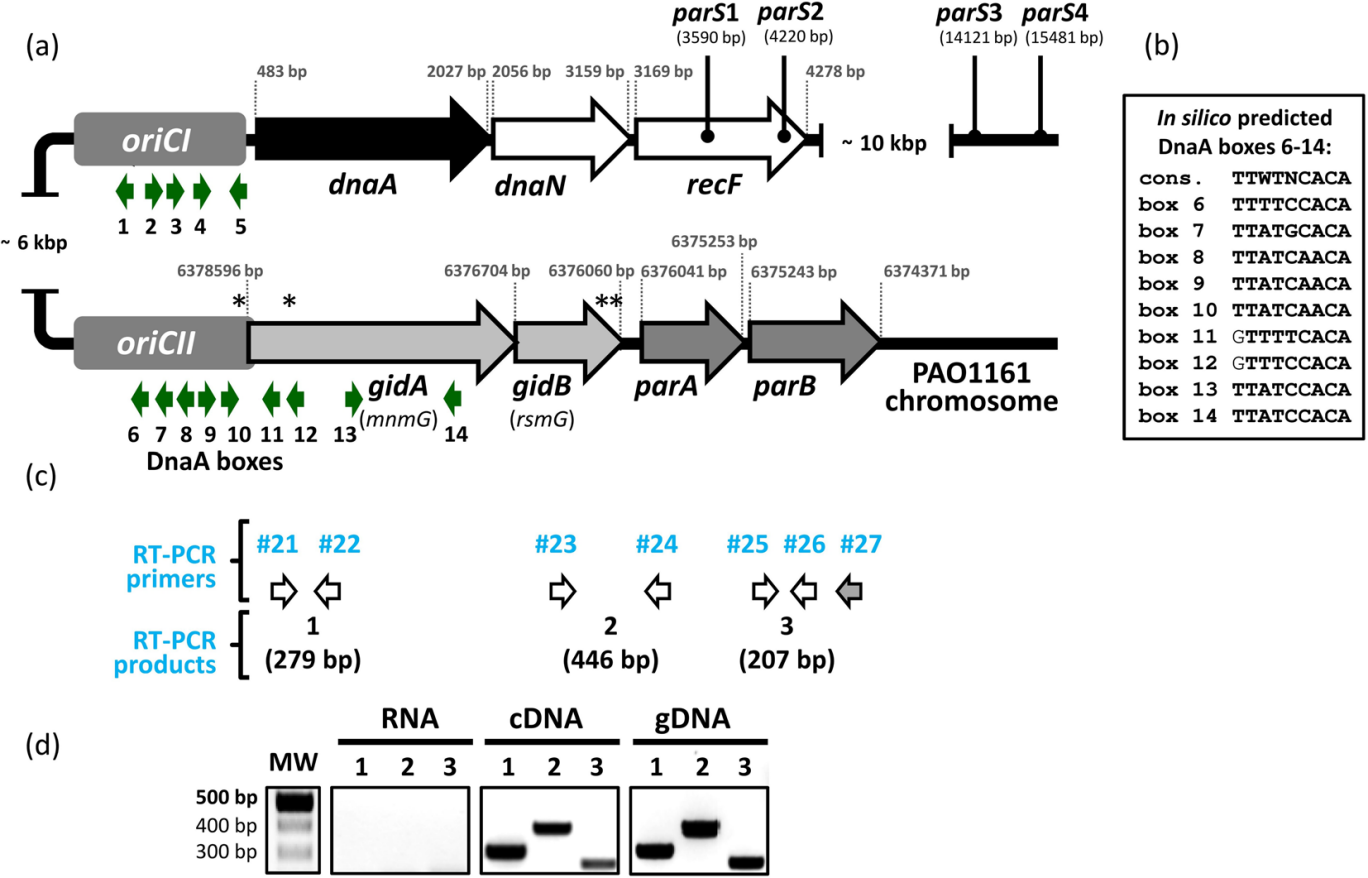
Verification of transcriptional coupling between *gid* and *par* genes. (**a**) Schematic representation of the *P. aeruginosa* PAO1161 (Acc. No. NZ_CP032126.1) chromosome fragment containing *oriC* region. DnaA boxes, marked by green arrows, were mapped based on our predictions and the results of Jiang et al., [28]. Transcriptional start sites (+1) predicted by dRNA-seq (BioSample: SAMN01084070) are indicated by an asterisk. (**b**) DNA sequences of in silico predicted DnaA boxes identified within *gidAB-parAB* operon. (**c**) DNA primers used in RT-PCR. The grey arrow indicates the location of the cDNA synthesis primer (#27) hybridization site. Hybridization sites of PCR primer pair #21 and #22 (RT-PCR product 1), #23 and #24 (RT-PCR product 2), and #25 and #26 (RT-PCR product 3) are indicated as open arrows. (**d**) DNA electrophoresis of RT-PCR products (1-3) with appropriate controls. DNA amplification was performed using: DNase treated RNA, cDNA, and genomic DNA as a template. The experiment was performed in a single replicate. Original gels are presented in Supplementary Fig. S7 online.

In *P. aeruginosa*, including the PAO1161 strain, *parA* and *parB* genes are located downstream of the *gidA* (*mnmG*) and *gidB* (*rsmG*) genes (Fig. 1a). Four copies of *parS* sequences (at least one of them indispensable for chromosome segregation) are located ~ 10 kbp (*parS1*-*2*), and ~ 20 kbp (*parS3*-*4*), from *parAB* genes, near the chromosome replication origin region, *oriCI* (Fig. 1a). *P. aeruginosa* is known to carry a second replication origin-like region, termed *oriCII*, which partially overlaps with the *gidA* gene^28^. The fragment encompassing the PAO1161 *oriCII* region was used to assess the presence and prevalence of *oriCII*, as well as its link with the *gidAB-parAB* loci in other *Pseudomonas* sp. In 725 sequenced *P. aeruginosa* strains, the *oriCII* region was identified with more than 96% identity to the corresponding region in the PAO1161 reference. This region co-occurs and overlaps with the 5’ end of the *gidA* gene, confirming the widespread occurrence and conservation of this locus among *P. aeruginosa* strains.

It was proposed that *oriCII* contains five DnaA box elements located upstream of the *gidA* gene^28^. Our predictions revealed four additional putative DnaA boxes. Two are positioned within the 5’ part of the *gidA* gene near *oriCII* (DnaA box 11 and 12), and two others are positioned in the central and 3’ part of the *gidA* gene (DnaA box 13 and 14) (Fig. 1a,b and Supplementary Fig. S1 online). Overall, there are a total of 129 in silico predicted DnaA boxes in PAO1161 genome, matching the consensus sequence TTWTNCACA, located both in coding and intergenic regions, with enrichment on the left chromosome arm (Supplementary Fig. S2 online, Supplementary Table S1 online). Given that DnaA can regulate gene expression, we hypothesized that the *gidAB*-*parAB* operon may be directly regulated by DnaA-interacting DnaA boxes within *oriCII*.

The proximity of the *gidAB* and *parAB* genes suggested that they might form an operon. To verify this, RT-PCR was performed using a cDNA template generated with a primer complementary to the *3’* part of the *parB* (oligo #27, Fig. 1c). Indeed, amplification of *gidA*, *gidB*, *parA*, and *parB* fragments, supports transcriptional continuity between these genes (Fig. 1d).

Interestingly, recent *P. aeruginosa* transcriptome analyses suggested three additional putative internal promoters within this operon. Based on dRNA-seq, four transcription start sites were predicted (our analyses of the data available under BioProject accession PRJNA169508 in the NCBI BioProject database). One located between DnaA box 10 and 11, the second within DnaA box 12, and two others in the terminal part of the *gidB* gene just upstream of *parA* (Fig. 1a). These predicted internal transcription start sites were therefore experimentally tested for promoter activity to estimate the scale of their contribution to the *parAB* gene expression.

### Identification of *gidAB-parAB* operon promoters

**Fig. 2:**
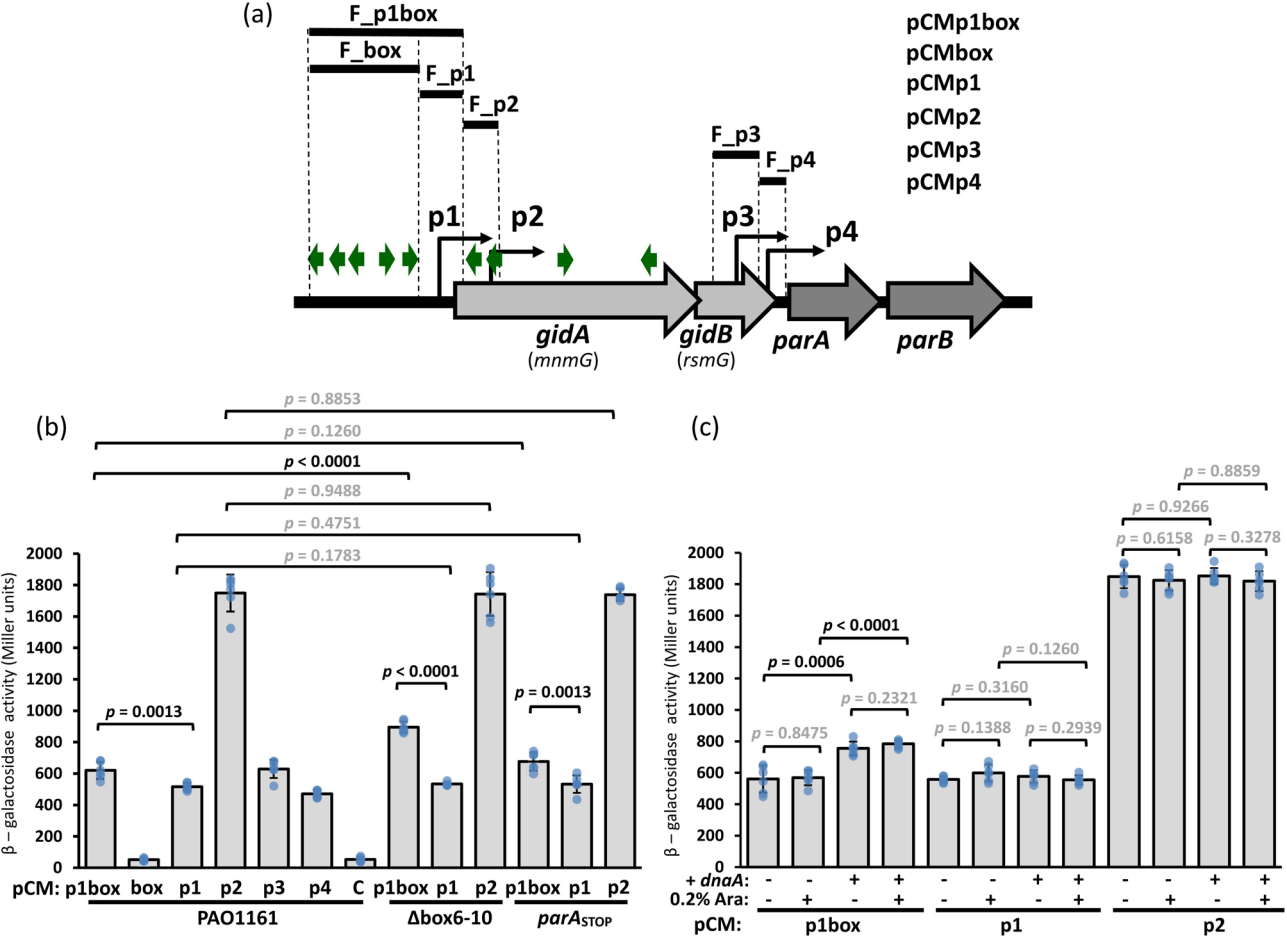
Promoter activity testing of DNA fragments from the *gidAB-parAB* operon. (**a**) Schematic representation of the *gidAB-parAB* operon. Green arrows represent DnaA box sequences. Putative promoter sequences (p1-p4) are indicated as black arrows. Black upper lines represent DNA fragments (F_p1box, F_box, F_p1, F_p2, F_p3, and F_p4) cloned in the promoter test vector pCM132 containing the promoter-less *lacZ* reporter gene. Obtained plasmid constructs were introduced into the PAO1161 strain or its derivatives, and promoter activity was measured using β-galactosidase as the reporter. (**b**) Promoter activity detected in PAO1161 WT harboring individual constructs. C represents pCM132 – “empty” vector used as a negative control. Promoter activity of F_p1 and F_p1box, as well as F_p2 DNA fragments cloned in pCMp1 and pCMp1box, and pCMp2 respectively, was also investigated in ∆box6-10 strain, PAO1161 with deletion of DnaA box sequences (see Fig. 4a). The p1 promoter activity was also detected in *parA*_STOP_ strains harboring pCMp1, pCMp1box, and pCMp2 plasmids. (**c**) Promoter activity of F_p1 and F_p1box, as well as F_p2 DNA fragments cloned in pCMp1, pCMp1box, and pCMp2 respectively and introduced into PAO1161 (WT) and I1A_*dnaA* strain (PAO1161 derivative with an additional chromosomal copy of a *dnaA* under the control of pBAD promoter, “+ *dnaA*”). Promoter activity was detected in bacterial cells cultured in an LB medium, and LB medium supplemented with 0.2 % arabinose for the *dnaA* gene overexpression. The experiment was performed in six biological replicates. Data represent mean ±SD and blue dots represent the individual measurements. Statistical significance was determined using an unpaired, two-tailed Student’s t-test (n = 6 per group). The threshold for statistical significance was set at α = 0.05, p-values (probability value) indicating significant differences (p < 0.005) are shown in black text.

The functionality of predicted promoters was verified by cloning the corresponding fragments in the pCM132 promoter test vector, carrying a promoter-less *lacZ* reporter gene (Fig. 2a and Supplementary Table S2 online), and β-galactosidase activity measurements upon introduction of the constructs into PAO1161 (Fig. 2b). The analysis indicated presence of promoter activity in fragments carrying the predicted p1- p4 regions (Fig. 2b). The highest activity, about 1750 Miller units, detected in the case of the p2 promoter was about three-fold higher than the one of p1, p3, and p4 promoters. Interestingly, p1 promoter activity was enhanced by the presence of the upstream region harbouring DnaA boxes 6–10 (fragment F_p1box, Fig. 2a). When coupled with this sequence, p1 activity increased from about 515 Miller units to about 620 Miller units. Consequently, the DNA fragment, containing only the DnaA-binding region (fragment F_box), had no promoter activity alone. Together, these results indicate that the *gidAB* and *parAB* can be transcribed from a common upstream promoter, while presence of additional internal promoters suggests a more complex regulatory scheme.

### Examination of the influence of DnaA on p1 and p2 activity

Extension of the p1 containing fragment by the upstream DnaA-binding region, enhanced its promoter activity in the test vector (Fig. 2b). This DNA segment contains putative DnaA boxes 6–10 (Fig. 1a), suggesting the possibility of the *gidAB-parAB* expression regulation by DnaA. To test this, promoter activity was analysed in PAO1161 derivative carrying an additional copy of the *dnaA* gene under the control of arabinose inducible p*BAD* promoter, inserted into the chromosome, allowing DnaA overproduction (strain I1A_*dnaA*). DnaA overproduction resulted in increased β-galactosidase activity in cells carrying pCM132 with the p1-box (harbouring DnaA boxes 6–10), but not for the p1 variant devoid of this fragment (Fig. 2c). Differences in promoter activity levels were evident even without arabinose-induced DnaA overexpression. This is due to the fact that the p*BAD* promoter is not as tightly controlled in *P. aeruginosa* cells as it is in *E. coli*, which limits the feasibility of controlling this promoter using either arabinose as an inducer or glucose as a repressor^33,34^.

Interestingly, a similar effect was observed when pCMp1box and pCMp1 were introduced into the PAO1161 ∆box6-10 strain, where five DnaA box sequences upstream of the *gidA* gene were deleted (Fig. 2b). One possible explanation is an increase in the available DnaA due to deletion of high-affinity binding sites; however, indirect effects cannot be excluded. Overall, both experimental approaches are consistent with a model in which DnaA availability modulates p1 promoter output. In contrast, DnaA abundance did not increase the β-galactosidase activity in cells carrying pCM132 with p2 (Fig. 2c), a fragment harbouring two elements resembling DnaA box sequences (box 11 and box 12).

Previous work indicated that the lack of *parA* protein causes the decrease in ParB level in *P. aeruginosa* cells^35^. Given that in some bacteria, the ParA protein stimulates the DnaA transcriptional regulator function^36^, we tested whether ParA influences DnaA-dependent regulation of the p1 and p2 promoter in *P*. *aeruginosa*. The analysis of promoter activity cloned in pCMp1box, pCMp1 as well as pCMp2 in PAO1161 and PAO1161 ParA deficient cells (*parA*_STOP_) did not however indicate a measurable change in promoter activity relative to parental (WT) strain (Fig. 2b).

**Fig. 3:**
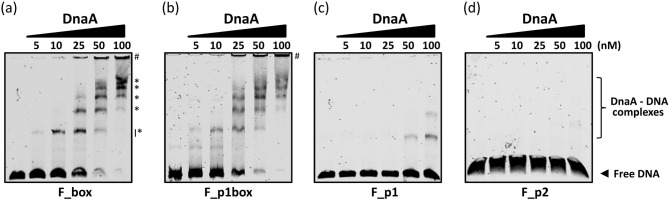
Electrophoretic mobility shift assay (EMSA). (**a**-**d**) show binding of purified DnaA protein to FAM-labeled DNA fragments of the *gidAB-parAB* operon (see Fig. 2a). Nucleoprotein complexes are marked with an asterisk, #DnaA-DNA complexes trapped in the loading wells, the result of DnaA oligomerization or aggregation (*P. aeruginosa* DnaA exhibits a high propensity for in vitro aggregation, even in the presence of high concentrations of bovine serum albumin, BSA). Original images are presented in Supplementary Fig. S8a online.

To investigate the direct interaction of the DnaA protein with DNA fragments containing the p1 and p2 promoters, an EMSA experiment was performed with purified *P*. *aeruginosa* DnaA protein (Supplementary Fig. S3 online). Four DNA regions tested previously in vivo were selected for analysis: full length p1 (F_p1box), p1 devoid of DnaA boxes 6–10 (F_p1), fragment carrying only DnaA boxes 6–10 (F_box), and p2 promoter with sequences closely resembling DnaA boxes (boxes 11 and 12) (F_p2). At lower concentrations (5 nM), DnaA formed a complex with both fragments containing DnaA boxes, marked as complex I* (Fig. 3a). At higher DnaA concentrations (25–100 nM) additional complexes of DnaA with the DNA fragments containing DnaA boxes 6–10 appeared, consistent with cooperative binding to multiple DnaA boxes (Fig. 3a, b). As expected, no interaction of DnaA was observed with the F-p1 fragment carrying p1 promoter deprived of DnaA boxes at lower DnaA concentrations (Fig. 3c). However, at higher DnaA concentrations (50–100 nM), additional complexes were also observed. Surprisingly, no detectable DnaA binding to the p2 promoter fragment was observed under the tested conditions despite the presence of two sequences closely resembling DnaA boxes in the tested fragment (Fig. 3d). Overall, the in vitro analysis indicated strong interactions of DnaA with DnaA boxes 6–10. These sites may therefore represent the primary high-affinity DnaA binding region within the *gidAB*-*parAB* promoter.

### Construction of deletion mutants in the *gidAB-parAB* region and their characteristics

**Fig. 4:**
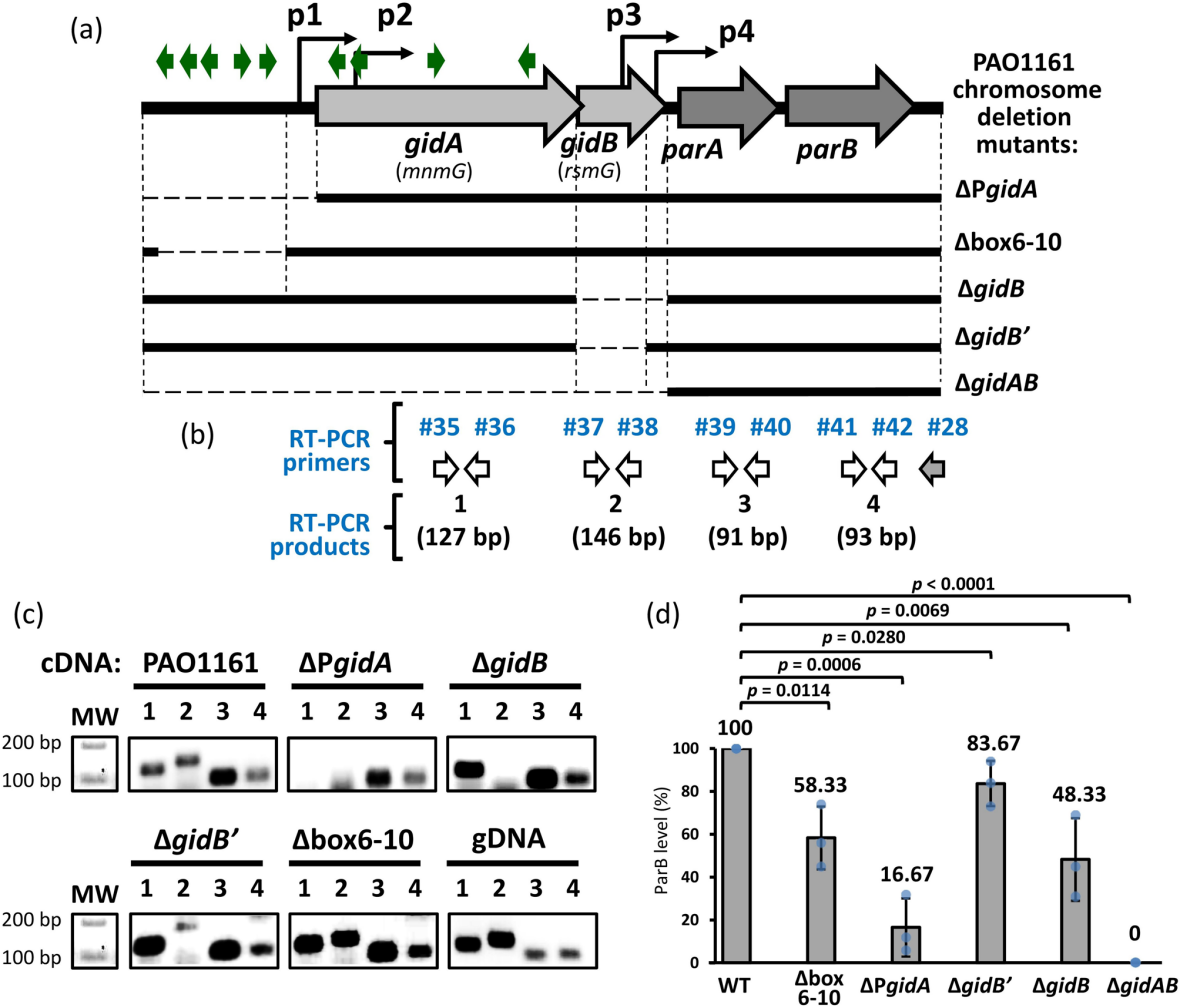
Functional analysis of the* gidAB-parAB* operon deletion mutants. (**a**) Schematic representation of the *gidAB-parAB* operon. The black lines below illustrate chromosome DNA fragments with deletion mutations (horizontal dashed lines) introduced in the chromosomes of the constructed strains. (**b**) RT-PCR analysis. The grey arrow indicates the location of the cDNA synthesis primer (#28) annealing site. Hybridization sites of PCR primer pair #35 and #36 (PCR 1), #37 and #38 (PCR 2), #39 and #40 (PCR 3), as well as #41 and #42 (PCR 4), are indicated as open arrows. (**c**) DNA electrophoresis of RT-PCR products (PCR with primer sets 1-4 from (**b**)), template cDNA was obtained from RNA transcripts from the PAO1161 (WT) strain and its deletion derivatives: ΔP*gidA*, Δ*gidB*, Δ*gidB*’, Δbox6-10; gDNA – genomic DNA used as a template in the control PCR reaction. Original gels are presented in Supplementary Fig. S9 online. (**d**) Detection of ParB protein level in PAO1161 and the constructed mutants by Western blot. Cells were collected, boiled in 1x SDS-PAGE loading buffer, and samples were separated by SDS-PAGE on 12% polyacrylamide gels. ParB was detected using anti-ParB polyclonal antibodies. Relative ParB level *gidAB-parAB* was detected in logarithmic phase of culture growth, in three independent experiments (values marked with dots on the bar chart). Sample volumes, loaded onto SDS-PAGE gel, were normalized according to the desired CFU number. The relative level of ParB was estimated densitometrically. Original blots are presented in Supplementary Fig. S5 online. Data represent mean ±SD and blue dots represent the individual measurements. Statistical significance was determined using an unpaired, two-tailed Student’s t-test (n = 3 per group). The threshold for statistical significance was set at α = 0.05), p-value (probability value).

To investigate the contribution of selected promoters and coding regions to the regulation and function of the *gidAB*-*parAB* operon, *P. aeruginosa* PAO1161 mutants, with deletions in the *gidAB-parAB* operon encompassing promoter regions, were constructed using the two-step allelic exchange method (for more details, see Methods section and Supplementary Table S2 online). Five PAO1161 derivatives were obtained (Fig. 4a): strain ∆P*gidA*, lacking the p1 promoter and DnaA boxes 6–10 located just upstream of the *gidA* gene; strain ∆box6-10, mentioned before, with deleted DnaA boxes 6–10; strain ∆*gidB* devoid of both p3 and p4 promoters positioned within the *gidB* gene; strain ∆*gidB’*, lacking p3 promoter; and strain ∆*gidAB*, with deletion of DNA fragment containing all four identified promoters and *gidAB* genes.

### Presence of transcripts for individual genes in *gidAB-parAB* operon mutants

The influence of introduced deletions on *gidAB-parAB* operon transcription was investigated using RT-PCR (Fig. 4b, c). A gene-specific primer (#28) was used for cDNA synthesis, and four primer pairs were used for the amplification of sequences corresponding to individual *gid* and *par* genes (Fig. 4b, c). In the case of the PAO1161 WT strain, all PCR products were detected, similarly, in the case of the ∆box6-10 strain. As expected, in ∆*gidB*, and ∆*gidB’*, there were no PCR products for the *gidB* gene-specific amplification, while other amplification products for *gidA*, *parA*, and *parB* were observed. In ∆*PgidA*, no RT-PCR products corresponding to *gidA* and *gidB* were detected, suggesting that the p2 promoter does not measurably contribute to *gidAB* transcription in ∆*PgidA* background. Nonetheless, transcripts for *parA* and *parB* were present in ∆*PgidA* mutant cells confirming the role of internal p3 and p4 promoters in *parAB* expression (Fig. 4c).

### The influence of promoter deletions on ParB levels from the *gidAB*-*parAB* locus

In the next step, the intracellular level of ParB, protein product from the last gene of the *gidAB*-*parAB* locus, was estimated in mutant strains compared with PAO1161 strain grown in rich LB medium, in the exponential phase of culture growth (Fig. 4d). We assumed that under these conditions, changes in ParB abundance likely reflect altered transcriptional input, although effects of altered protein stability cannot be excluded. Crude bacterial lysates obtained from the same cell number were loaded onto SDS-PAGE gel, and ParB was detected by Western blot with anti-ParB antibodies. The ParB level was estimated by the densitometric method (see Supplementary Fig. S5 online). The analysis showed that deletion of the p1 promoter in ∆*PgidA* resulted in a decrease in the ParB abundance to about 16% of the WT strain level (Fig. 4d). Similarly, removal of the DnaA boxes 6–10 region (∆box6-10 strain) led to a 40% decrease of the ParB level. In contrast, deletion of the p3 promoter (∆*gidB’* strain) had only a minor effect on the ParB level. Concomitantly, lack of p3 and p4 promoters (∆*gidB* mutant) resulted in about 50% of native ParB level in the cells. Finally, in the case of the ∆*gidAB* strain, no ParB was detected, suggesting there are no *parB* promoters within a *parA* gene or in the region upstream from the p1 promoter (Fig. 4d). Taken together, these results demonstrate that the *gidAB-parAB* gene cluster forms a complex operon, with internal p3 and p4 promoters that allow independent expression of *parAB* even when the p1 promoter is disrupted.

### The influence of *gidAB-parAB* operon mutations on streptomycin susceptibility

**Fig. 5:**
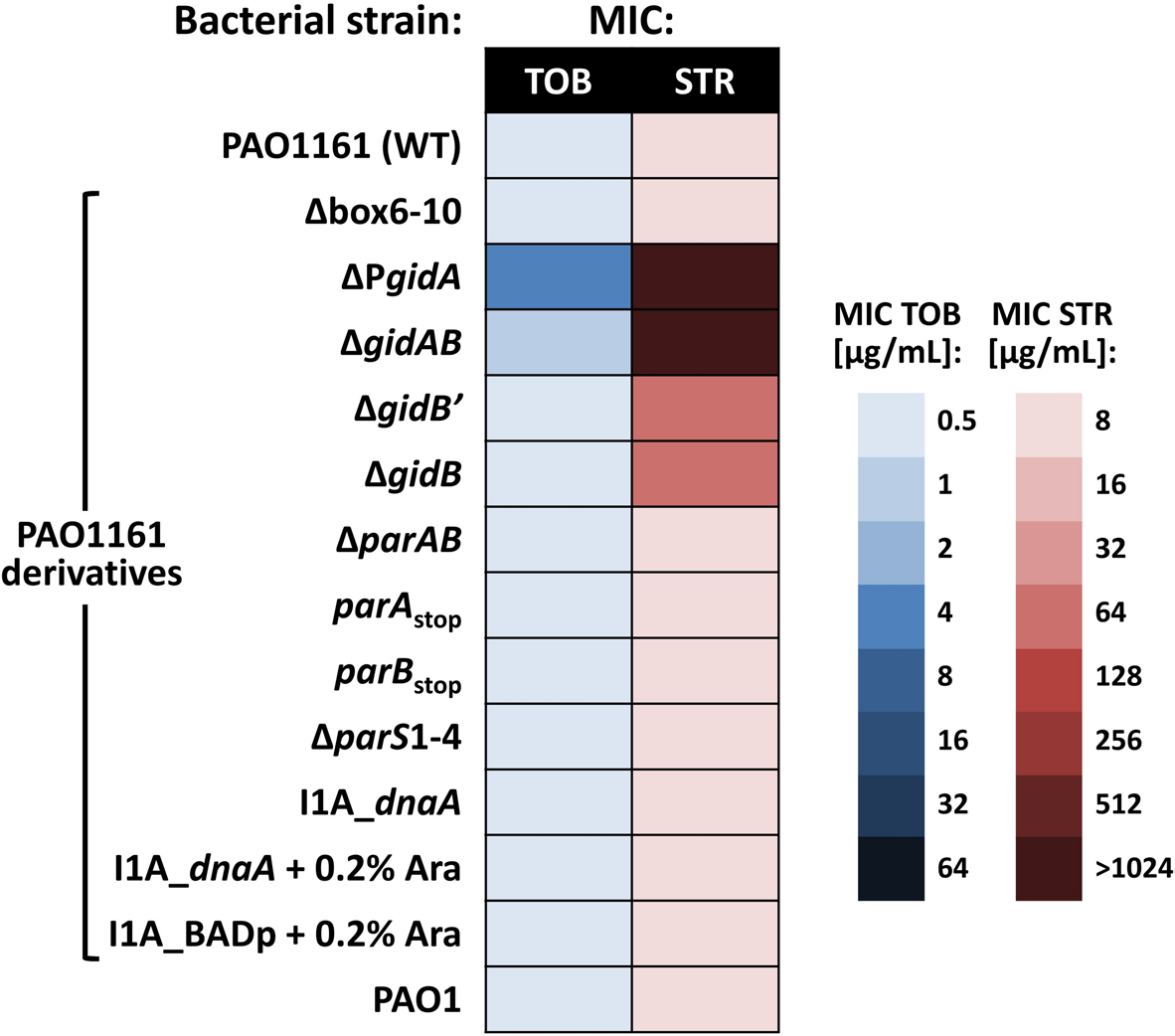
Effect of mutations within the *gidAB*-*parAB* operon on antibiotic resistance. Minimum inhibitory concentrations (MICs) of streptomycin (STR) and tobramycin (TOB) were determined by the broth microdilution method. For each strain 3 biological replicates were routinely used and experiment was repeated twice, with the same results. Data represent median from 3 biological repeats. I1A_*dnaA* – PAO1161 derivative carrying an additional copy of the *dnaA* gene under the control of arabinose–inducible pBAD promoter, inserted into the chromosome; I1A_BADp – PAO1161 with “empty” genetic cassette containing pBAD promoter inserted into the chromosome; PAO1 – *P. aeruginosa* reference strain (Supplementary Table S3 online); + 0.2% Ara – arabinose supplementation.

Mutations in *gidB* are known to confer increased streptomycin resistance in several pathogenic bacteria^23,24^, but this phenotype has not been examined in *P. aeruginosa*. To address this, we analyzed the susceptibility of *gidAB*-*parAB* operon mutants to two aminoglycosides, streptomycin and tobramycin (Fig. 5). The parental PAO1161 strain exhibited growth up to 4 µg/mL streptomycin, corresponding to a minimum inhibitory concentration (MIC) of 8 µg/mL. Inactivation of *gidB* increased streptomycin resistance, with the MIC rising to 64 µg/mL in the Δ*gidB* and Δ*gidB*′ mutants and to > 1024 µg/mL in the Δ*gidAB* and ΔP*gidA* strains. In contrast, no changes in streptomycin MIC were observed for the Δbox6–10 strain and Δ*parAB* mutant with *parAB* operon deleted (Fig. 5). Concomitantly, only a modest, twofold increase in MIC of another aminoglycoside - tobramycin was observed in the Δ*gidAB* and ΔP*gidA* mutants (Fig. 5), suggesting that the effect is likely specific to streptomycin.

To complement the MIC analysis and capture more subtle differences in streptomycin response, strain growth was monitored in the presence of a subinhibitory concentration of streptomycin (6 µg/mL). Under these conditions, the ΔP*gidA* and Δ*gidB* again showed improved growth relative to PAO1161, confirming their elevated streptomycin tolerance (Supplementary Figure S6 online). Under the same conditions, the Δ*parAB* mutant showed a growth delay (Supplementary Figure S6). To determine whether this sensitivity reflected disruption of the ParAB–*parS* system, strains carrying premature stop codons in *parA* (*parA*_STOP_) or *parB* (*parB*_STOP_), as well as the Δ*parS1*-*4* mutant, were examined. All three displayed the same streptomycin MIC as the parental PAO1161 strain and the Δ*parAB* mutant (8 µg/mL; Fig. 5), although their growth was not different or only modestly reduced in the presence of subinhibitory streptomycin compared with PAO1161 (Supplementary Figure S6 online). The increased sensitivity of the Δ*parAB* mutant may therefore result from a polar effect, e.g. on the downstream ATP synthase operon (*atpIBEFHAGDC*), which has been implicated in aminoglycoside resistance^37^, rather than from loss of ParAB–*parS* system function itself. Altogether, these results indicate that the increase in streptomycin tolerance is driven by disruption of *gidB* or its expression.

## Discussion

The genetic organization of bacterial chromosome replication origin is highly conserved and reflects the tight coordination required between DNA replication, chromosome segregation, and cell cycle progression^16,17^. In *P*. *aeruginosa*, as in many other bacteria, *oriC* domain contains *oriCI* with canonical *rnpA*-*rpmH*-*dnaA*-*dnaN*-*recF*-*gyrB*s gene cluster, but also *gidAB*-*parAB* operon located upstream with *oriCII*^17,28^. DnaA protein is the master initiator of chromosomal DNA replication, which has also been shown in several bacteria to function as a transcriptional regulator^1^. In this study, we demonstrate that in *P. aeruginosa* the *gidAB* and *parAB* genes form a single transcriptional unit directly regulated by DnaA through binding to DnaA boxes located within the *oriCII* region upstream of the *gidA*. This suggests a direct regulatory link between the replication initiation machinery and genes encoding key factors involved in cell growth and chromosome segregation.

**Fig. 6:**
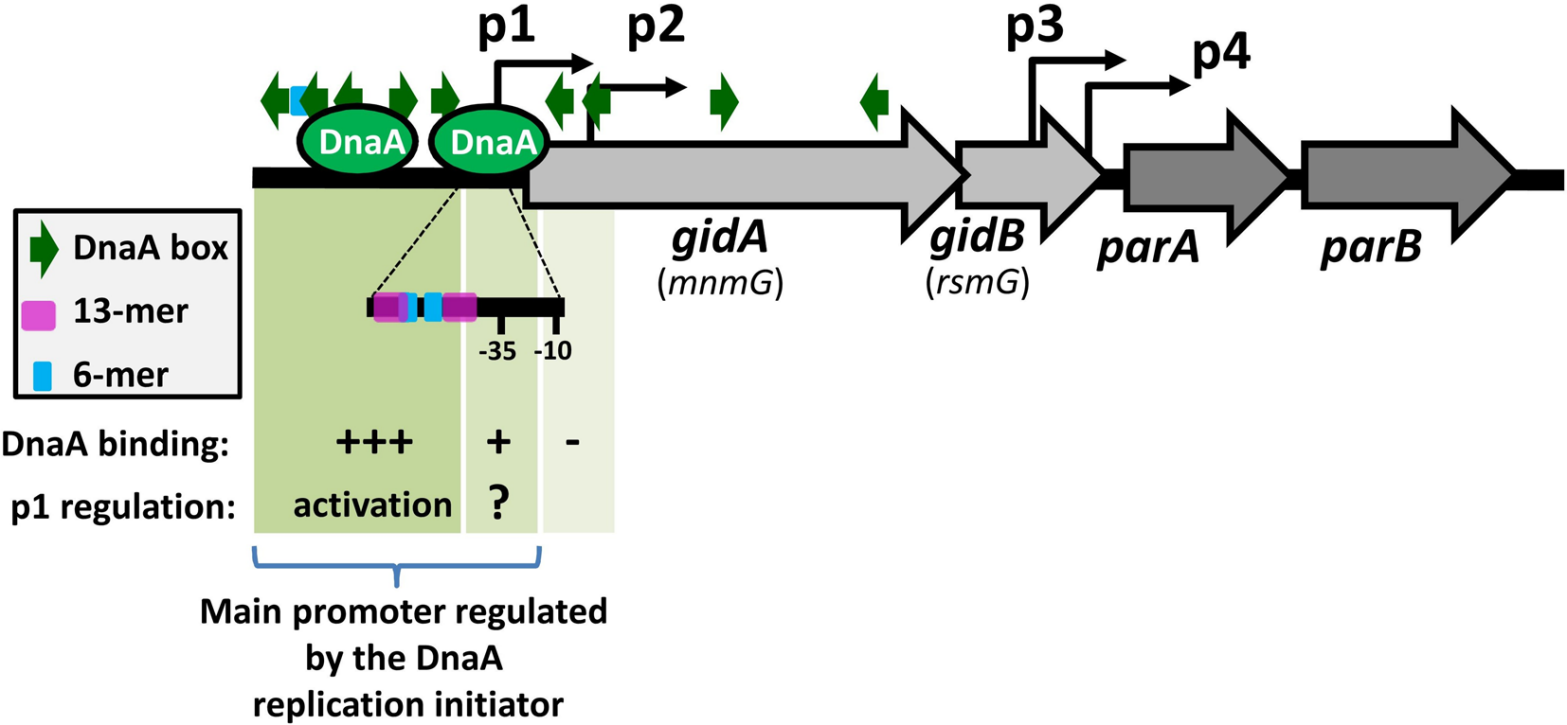
Regulatory elements controlling transcription of the *gidAB-parAB* complex operon.

Our data, in line with previous observations from the dRNA-seq, confirmed that transcription of the *gidAB-parAB* operon is driven by a complex promoter architecture consisting of one upstream promoter and three internal promoters. The primary promoter, named p1, is located just upstream of the *gidA* gene and overlaps the *oriCII* region, whereas promoter p2 lies within the 5’ part of the *gidA* coding sequence, and promoters p3 and p4 are located in the 3’ part of the *gidB* gene (Fig. 6). RT-PCR analysis revealed that in the case of the ΔP*gidA* strain (lacking p1), transcripts of *gidAB* genes, but not of *parAB* genes, were not detected (Fig. 4c), indicating that in this context p2, located in the 5’ part of *gidA* was inactive. This is in contrast to promoter-reporter assays, demonstrating the highest activity for p2 out of the tested fragments, however this discrepancy can likely be explained by differences between multicopy plasmid-based reporter assays and the native chromosomal context, where promoter accessibility, local DNA topology, and regulatory protein binding may constrain p2 activity. In contrast, transcription from the internal promoters p3 and p4 persisted in ΔP*gidA*, as evidenced by detection of *parA* and *parB* transcripts (Fig. 4c), although their contribution to overall ParB production was limited (roughly ~ 20%). Thus despite *parAB* having their own promoters, promoter p1 appears to represent the dominant upstream promoter contributing to *gidAB* and *parAB* expression in the chromosomal context (Fig. 6).

Detailed analysis of the region upstream of *gidA* revealed the presence of multiple putative DnaA binding sites (Fig. 1a, b). This *oriC*-like region (*oriCII*), previously shown to support autonomous replication when cloned on a plasmid, is conserved among *Pseudomonas* species and other members of the *Pseudomonadaceae*, although it is dispensable for chromosomal replication initiation in *P. aeruginosa*^28,38^. In addition to the five DnaA boxes previously reported in this region, our in silico predictions identified four additional sequences matching or closely resembling the DnaA box consensus TTWTNCACA, resulting in a dense cluster of potential DnaA binding sites upstream of the p1 promoter. Notably, five of these DnaA boxes (boxes 6–10) are positioned upstream of the *gidA* transcriptional start site (Fig. 6).

The closest to the start codon of the *gidA* gene is DnaA box 10, which is located 205 bp upstream, and 130 bp upstream from the predicted − 35 box, a core element of the promoter important for the recruitment of RNA polymerase^39^. Interestingly, DnaA box 10 together with the next closest DnaA box 9 are the only not reverse-oriented DnaA boxes in the promoter region of the *gidAB*-*parAB* operon, while DnaA boxes 6 and 7 perfectly match the TTWTNCACA consensus sequence for DnaA box^1,40^. Such an arrangement of DnaA boxes in the *gidA* promoter region is consistent with a positive regulatory role of DnaA in the activity of p1 promoter as indicated in reporter gene assay (Fig. 2). This array of DnaA boxes in the p1 promoter (F_p1box and F_box) was efficiently bound by DnaA in vitro in a concentration-dependent manner (Fig. 3). Since the first DnaA box in the p1 promoter sequence is located at a distance (130 bp) from the predicted − 35 box, it is tempting to speculate that long-range interactions within the promoter are involved in transcription activation with the participation of DnaA. Functional relevance of this DnaA box cluster was supported by promoter-reporter assays, in which extension of the p1 promoter fragment by the upstream DnaA box–containing sequence (F_p1box) resulted in a reproducible increase in promoter activity compared to the p1 fragment lacking this region (Fig. 2b). Consistently, deletion of DnaA boxes 6–10 from the chromosome (∆box6-10 strain) led to a substantial reduction in ParB protein levels, as detected by Western blot analysis (Fig. 4d), indicating that this conserved *oriCII* region contributes positively to the *gidAB–parAB* operon expression (Fig. 6).

The contribution of the DnaA box cluster to p1 promoter activity prompted us to examine whether DnaA directly interacts with this region and modulates transcription of the *gidAB-parAB* operon. In vivo, increased availability of DnaA in the I1A_*dnaA* strain resulted in enhanced activity of the p1 promoter fragment containing DnaA boxes 6–10 (F_p1box), whereas no such effect was observed for the p1 fragment lacking this region (Fig. 2c). This observation is consistent with a positive regulatory role of DnaA acting through the upstream DnaA box cluster. A similar increase in p1-driven reporter activity was also detected in the ∆box6-10 strain (Fig. 2b), likely reflecting increased intracellular availability of DnaA upon deletion of high-affinity binding sites, although indirect effects cannot be excluded.

Direct interaction of DnaA with the p1 regulatory region was confirmed by electrophoretic mobility shift assays using purified *P. aeruginosa* DnaA protein (Fig. 3). DnaA formed stable nucleoprotein complexes with DNA fragments containing DnaA boxes 6–10 (F_p1box and F_box) at low protein concentrations, whereas binding to the p1 fragment lacking these boxes (F_p1) was observed only at higher DnaA concentrations and likely reflects nonspecific interactions (Fig. 3a–c). Another possibility is that DnaA binds to 6mer-like sequences located near, or within 13-mers present within F_p1 (Supplementary Fig. S1 online). Both resemble consensus sequence AGATCT of ATP-DnaA boxes^5^. However, it was postulated that this type of sequence is recognized by the DnaA only if the adjacent 9mer DnaA box is present^5^. Two additional putative low-affinity DnaA boxes 11 and 12 (none of them perfectly matching the consensus sequence of DnaA box), located within the *gidA* coding sequence, were not bound by DnaA under the tested conditions in EMSA (F_p2). Both are reverse-oriented relative to the *gidA* gene, with an 18-bp distance between. Furthermore, the analysis of the effect of DnaA on p2 transcriptional activity did not confirm that this protein acts as a regulator of p2 (Fig. 2b, c). However, we cannot rule out that DnaA interacts with them when other boxes (e.g. 6–10), present *in cis*, are filled during cooperative DnaA binding^41^. Together, these data suggest that DnaA directly and preferentially binds to the *oriCII* located DnaA box cluster upstream of *gidA*, supporting a model in which DnaA positively regulates p1 promoter activity through direct interaction with this region (Fig. 6).

Genes encoding GidA and GidB proteins are located in proximity to DnaA box regions in genomes of different bacteria^42^. However, regulation of the *gidAB* genes by DnaA protein has not been widely reported. Weak, but statistically significant repression of *thdF*-*gidA*-*gidB*-*noc* operon was observed in *B. subtilis* cells overproducing DnaA^43^, which is in contrast to DnaA-mediated regulation mode identified here for *P. aeruginosa*. These differences may reflect distinct genetic organization of the operons or species-specific regulatory mechanisms^43^. Notably, DnaA has been shown to act both as a transcriptional activator and repressor, depending on its concentration and nucleotide-bound state, as shown e.g. for the *nrdAB* operon in *E. coli*^15^. In this context, our findings extend the known spectrum of DnaA regulatory activities and identify the *gidAB-parAB* as a direct DnaA-regulated operon in *P. aeruginosa*.

ParB, acting together with its cognate ATPase ParA, and encoded within the *gidAB*-*parAB* operon, is responsible for the proper segregation of the bacterial chromosomes in *P. aeruginosa* and other bacteria^35,42,44^. Because this process must be strictly regulated and coordinated with DNA replication and cell division, regulatory cross-talk between ParAB-*parSs* and proteins involved in replication initiation has emerged^25^. Indeed, direct interactions between ParB and DnaA protein were demonstrated in several species including *Deinococcus radiodurans*^45^ and *Vibrio cholerae*^46^, and have been implicated in modulation of replication initiation or transcriptional regulation. In *B. subtilis*, ParB (Spo0J) influences both transcription regulation and replication initiation activities of DnaA indirectly, by modulation of ParA (Soj) ATPase activity, while only Soj interacts with the DnaA protein^36,47^. In *P. aeruginosa*, our data indicate that such coordination is achieved primarily at the transcriptional level, through DnaA-dependent activation of the p1 promoter driving expression of the entire *gidAB-parAB* operon. At the same time, the presence of internal promoters p3 and p4 within the operon allows continued, albeit significantly reduced, expression of *parAB* when p1 activity is compromised, as observed in the ΔP*gidA* background (Fig. 4c, d). This promoter organization suggests a regulatory architecture that couples chromosome segregation to replication initiation while preserving a degree of transcriptional robustness necessary for maintenance of essential ParA and ParB levels. In both WT and *parA*_STOP_ mutant strains, the p1 promoter of the *gidAB*-*parAB* operon appears to be regulated by DnaA in the same way (Fig. 2b). Further investigations are required to conclusively determine whether ParA in *P. aeruginosa* modulates the activity of DnaA under specific conditions.

In many organisms, DnaA regulates its own expression, forming a feedback loop that ensures appropriate intracellular levels of the protein for replication initiation and transcriptional regulation^5,11,48^. Moreover, DnaA regulons have been described in several bacterial species. In *C. crescentus*, DnaA coordinates DNA replication initiation with cell cycle progression^49^, while in *B. subtilis*, DnaA influences the expression of multiple regulons^1,5,10,48,50^. Moreover, DnaA-dependent regulation extends to the *E. coli* SOS regulon^51^. To our knowledge, this study represents the first experimental demonstration of direct DnaA-dependent regulation of a chromosomal operon in *Pseudomonas* spp. Previous studies in *P. putida* did not detect DnaA autoregulatory activity^52^, highlighting potential species-specific differences in DnaA-mediated transcriptional control. In *P*. *aeruginosa*, direct transcriptional regulation by DnaA has so far been experimentally confirmed only for the *gidAB-parAB* operon described here. Importantly, it is known that *P. aeruginosa* GidA influences the expression of numerous genes at the post-transcriptional level^20,53^, and our findings indicate that *P. aeruginosa* ParB is a multifunctional protein that may also contribute to regulation of gene expression^54,55^. Taken together, these observations suggest that DnaA can exert broader regulatory effects in *P. aeruginosa* indirectly, through modulation of the *gidAB*-*parAB* operon transcription. Moreover, the presence of multiple putative DnaA-binding sites across the *P. aeruginosa* chromosome (Supplementary Fig. S2 and Table S1 online) points to a potential further involvement of DnaA in gene expression regulation, a possibility that will require further experimental analysis.

Strains lacking the p1 promoter (ΔP*gidA*) or deletion encompassing p1 together with both *gidA* and *gidB* genes (Δ*gidAB*) showed growth defects, most likely resulting from the absence of the GidA protein, which is known to be critical for cellular physiology^20^. Phenotypes of single mutations of individual genes of the *P. aeruginosa gidAB*-*parAB* operon have been reported previously except for the *gidB* gene^20,35,45,55,56^. Deletions within the *gidB* gene (∆*gidB* and ∆*gidB’*) did not have a significant effect on bacterial growth on rich medium under non-stress conditions (Supplementary Fig. S4 online), in line with observations in *Salmonella typhimurium*^23^, *B. subtilis*^57^. However, inactivation of *gidB* gene has been reported to confer streptomycin resistance in a variety of bacterial species, including low level resistance in *Mycobacterium tuberculosis* and *Streptomyces coelicolor*^57–60^; intrinsic streptomycin resistance in *Bacillus velezensis*^24^, or high-level antimicrobial resistance in *Salmonella*^23^. GidB functions as 16 S rRNA methyltransferase, catalyzing methylation of guanosine 527, and mutations that disrupt S-adenosylmethionine binding or reduce methyltransferase activity lead to streptomycin resistance as reduced methylation of 16 S rRNA decreases streptomycin binding to the ribosome^57,59^. Consistent with these observations, deletion of *gidB* in *P. aeruginosa* resulted in a high level of streptomycin resistance (Fig. 5). Surprisingly, an even greater effect was observed in the case of the ∆P*gidA* and ∆*gidAB* strains. Most likely this is due to the lack of GidB and GidA, or the reduction of both protein levels. Recently, it was reported that *gidA* gene deletion (most likely through an indirect mechanism) results in increased tolerance to a number of different types of antibiotics, including aminoglycoside antibiotics (e.g. tobramycin)^61^. This is confirmed by the observation that ∆P*gidA* and ∆*gidAB* mutants show increased resistance to both tobramycin and streptomycin, whereas ∆*gidB’* and ∆*gidB* mutants show increased resistance only to streptomycin (Fig. 5). Deletion of box6-10 sequences, while also negatively affecting the expression of the *gidAB*-*parAB* operon, does not cause streptomycin or tobramycin resistance (Fig. 5). Nonetheless, expression of *gidAB* is not inhibited in the ∆box6-10 strain (Fig. 4c), and GidAB level in ∆box6-10 can be sufficient to maintain the WT strain phenotype.

Summarizing, in this work we demonstrate direct transcriptional control of the *gidAB*-*parAB* operon by DnaA in *P. aeruginosa*, providing another example documenting the dual role that DnaA plays in bacterial cells, as a replication initiation factor, but also as a transcriptional regulator. This dual function enables DnaA to coordinate DNA replication with other essential cellular processes, including chromosome segregation, cell growth, and antibiotic susceptibility as exemplified in the proposed model (Fig. 6). Identification of the regulatory elements controlling *gidAB-parAB* expression, together with demonstration of the link between *gidB* disruption and streptomycin resistance, underscores the clinical relevance of this regulatory network in *P. aeruginosa*.

## Methods

### Bacterial strains, plasmids, and culture conditions

Bacterial plasmids and strains used in this study are listed in Supplementary Tables S2 and S3 online. All strains were cultured at 37 °C in Luria-Bertani broth (LB) medium (tryptone 10.0 g/L, yeast extract 5.0 g/L, and NaCl 5.0 g/L; pH 7.2–7.5), or cation-adjusted MH broth, (Mueller-Hinton II, BD Difco). When necessary, the medium was supplemented with appropriate antibiotics at the following concentrations, for *E. coli*: ampicillin (Amp) – 100 µg/mL, kanamycin (Kan) – 50 µg/mL, or chloramphenicol (Chl) – 34 µg/mL; for *P. aeruginosa*: rifampicin (Rif) − 300 µg/mL, carbenicillin (Car) – 300 µg/mL, kanamycin (Kan) – 500 µg/mL, streptomycin (Str) – 6–1024 µg/mL, tobramycin – 0.5–64 µg/mL, or chloramphenicol (Chl) – 150 µg/mL. For induction of p*BAD* promoter-dependent protein overexpression, arabinose was added to the medium at a final concentration of 0.2%. Bacterial cells were routinely grown in flasks sealed with a cotton plug, with shaking at 200 rpm at 37 °C. Growth kinetics were monitored by measurements of optical density at 600 nm (OD600) in 96-well plates at 37 °C using a Varioskan Lux Multimode Microplate Reader and SkanIt RE 7.0.2.5 software (Thermo Fisher Scientific, Waltham, MA, USA). Overnight cultures were diluted 1:200 in fresh LB medium, and 200 µl culture aliquots were transferred to 96-well microtiter plates. The plates were incubated at 37 °C with shaking (in LB or cation-adjusted MH broth), and OD600 was measured at 15 min intervals.

### Construction of *P. aeruginosa* deletion mutants by homologous recombination

The pAKE600 suicide vector was used to prepare alleles of genes of interest (Supplementary Table S2 online) and deletion mutants were obtained as described earlier^56^. Obtained clones, capable of growing on L-agar with 10% sucrose, and carbenicillin-sensitive strains, grown on such plates, were subjected to PCR to screen for colonies with exchanged allele. Primers used for strain verification were designed to anneal to the regions outside the sequences used as the regions of homology.

### Construction of promoter-*lacZ* transcriptional fusions and promoter activity testing

To test promoter activity in *P. aeruginosa*, a RK2 derivative, the pCM132 plasmid with a promoterless *lacZ* reporter gene was used (Supplementary Table S2 online). To construct pCM132 derivatives carrying predicted promoters from the *gidAB*-*parAB* operon, DNA fragments were amplified by PCR, digested by appropriate restriction enzymes, and ligated with cut pCM132 vector. Ligation products were introduced into *E. coli* S17-1 by chemical transformation, and plasmids were transferred to PAO1161 using conjugation. β-galactosidase activity assay was performed according to a method described by Thibodeau and co-workers^62^. The bacterial cultures were inoculated into 96-well microtiter plates in eight replicates in LB medium supplemented with kanamycin; when required, arabinose was added to a final concentration of 0.2%. Cultures were incubated for 4 h. Following the enzymatic reaction, the plate was placed in a Varioskan Lux Multimode Microplate Reader (Thermo Fisher Scientific, Waltham, MA, USA) and incubated at 28 °C, while absorbance at 415 nm was measured at 30-second intervals. The experiment was performed in six biological replicates.

### RT-PCR

For RNA isolation, overnight bacterial cultures of PAO1161 were diluted 1:50 in fresh LB medium and cultivated at 37 °C and 200 rpm. Culture samples were collected after 4 h (logarithmic phase). The cells were harvested by centrifugation, and total RNA was isolated from the pellet using a Total RNA Mini Plus kit (A&A Biotechnology), and treated with DNase using a Clean-Up RNA Concentrator kit (A&A Biotechnology). The quality and concentration of the isolated RNA were evaluated using a Picodrop Microliter UV/Vis Spectrophotometer. For cDNA synthesis, 1 µg of each total RNA sample was used in reaction performed at 65 °C using Maxima H Minus First Strand cDNA Synthesis Kit, with dsDNase (Thermo Scientific). For RT-PCR cDNA synthesis gene specific primers were used (Figs. 1c and 4b). cDNA samples were immediately used for PCR (Figs. 1d and 4c, and Supplementary Table S4 online). The experiment was performed in a single replicate.

### Western blot

Protein samples were separated on 12% polyacrylamide SDS-PAGE gels. After electrophoresis, the gel was incubated in a transfer buffer (1x SDS-PAGE buffer (25 mM Tris base, 200 mM glycine, 0.1% (w/v) SDS), and 20% methanol) for 10 min. Nitrocellulose Blotting Membrane “Protran” 0.45 μm NC (Amersham, 10600002, Buckinghamshire, UK) was incubated in a transfer buffer for 10 min. Proteins were transferred from the gel onto the membrane using a VWR “PerfectBlue” Semi-Dry Electro Blotter Sedec M device (VWR, Radnor, PA, USA) with transfer buffer (1x SDS-PAGE buffer, methanol 20% (v/v)) at 55 mA (1 mA/cm^2^) for 90 min. The transfer membrane was then incubated for 30 min in a blocking buffer (20 mM Tris, 150 mM NaCl, 0.05% Tween 20 and 5% nonfat-dried milk; pH 7.5) at room temperature. The blocked membrane was incubated with rabbit anti-ParB antibody^63^ for 2 h at room temperature. Next, the membrane was washed 3 times for 20 min in TBST buffer (20 mM Tris, 150 mM NaCl, 0.05% Tween 20; pH 7.5). The membrane was then incubated with Anti-Rabbit IgG (Fc) AP Conjugate antibodies (1:10000 dilution) (Promega, S3738, Madison, WI, USA) for 1 h at room temperature. After a further two 5 min washes in TBS, immunoreactive bands on the blot were detected using the BCIP/NBT (5-bromo-4-chloro-3-indolyl-phosphate/nitro blue tetrazolium) Color Development Substrate (Promega, S3771, Madison, WI, USA), according to the manufacturer’s instructions.

### Estimation of relative ParB level

To determine ParB level in *P. aeruginosa* cells, Western blot analysis combined with densitometric analysis was used. Culture samples were collected, harvested by centrifugation, resuspended in a 1x SDS-PAGE loading buffer (62.5 mM Tris-HCl (pH 6.8), 2% (w/v) SDS, 10% (v/v) Glycerol, 0.01% (w/v) Bromophenol Blue, and 5% β-mercaptoethanol), and boiled in a water bath. For each sample, a small aliquot was used to estimate the number of CFU ml^− 1^ by plating an appropriate dilution of the culture on LB agar plates. Sample volumes corresponding to the desired CFU number were separated by SDS-PAGE on 12% polyacrylamide gels. Separated proteins were transferred onto nitrocellulose membranes and visualized (Supplementary Fig. S5 online) according to the Western blot procedure described above. Band intensity was densitometrically estimated using ImageJ software. The experiment was performed in three biological replicates.

### DnaA protein overexpression and purification

*P. aeruginosa* DnaA protein was overexpressed from pET28-MBP-dnaA construct, in fusion with a His6-MBP-TEV tandem tag, consisting of hexahistidine peptide, maltose binding protein (MBP) sequence and TEV protease cleavage site. The MBP tag was employed to increase *P. aeruginosa* DnaA solubility. *E. coli* BL21(DE3) Δ*clpP* strain (Supplementary Table S3 online) was used as a host, to reduce DnaA proteolysis. The DnaA protein was purified using a three-step protocol involving a HisTrap HP column (5 mL, GE Healthcare) purification, a HiTrap Heparin HP column (5 mL, GE Healthcare) purification, and a second HisTrap HP column (5 mL, GE Healthcare) purification for removal of the His₆–MBP tag following TEV protease cleavage. The method is described in detail in the Supplementary Information (Supplementary Fig. S3 online).

### Electrophoretic mobility shift assay (EMSA)

EMSA was performed essentially as described earlier^64^, with minor changes. Briefly, PCR fragments F_p1box, F_p1, F_box, F_p2 were prepared using plasmids pUC-p1box, pUC-p1, pUC-box, pUC-p2 (Supplementary Table S2 online) as a template and vector-specific primers #45 (5’-6-FAM labeled) and #46 (Supplementary Table S4 online) flanking the fragments’ insertion site. DNA fragments (8 nM) were incubated with increasing DnaA concentrations (0-100 nM) in a total reaction volume of 20 µL containing 20 mM Tris-HCl, pH 7.6, 120 µg/mL bovine serum albumin, 4% sucrose, 4 mM dithiothreitol, 32 mM HEPES/KOH, 8 mM magnesium acetate, 2 mM ATP, 20 µg/ml poly(dI-dC), 0.08% Igepal CA-630. Nucleoprotein complexes were formed during 20 min incubation at 32 °C, subsequently 2.5 µL of 20% Ficoll was added to each sample and the samples were loaded on a 5% native acrylamide gel (acrylamide: bis 37.5:1). DnaA-DNA complexes were separated at 4 °C, in 0.5xTBE at approximately 10 V/cm of gel length. Prior to sample loading a pre-run was carried out under the same conditions for about 1 h. DNA was visualized using an Amersham Typhoon scanner (Cytiva) with excitation and emission wavelengths adjusted to the 6-carboxyfluorescein (6-FAM) spectrum (excitation at 488 nm; emission filter Cy2, 525BP20, 515–535 nm). The experiment was performed in two replicates.

### Determination of susceptibility to streptomycin

Minimum inhibitory concentrations (MICs) of streptomycin and tobramycin were determined by the broth microdilution method. Bacterial strains were cultured overnight in cation-adjusted MH broth, (Mueller-Hinton II, BD Difco) and diluted in fresh Mueller-Hinton II broth to a final density of 5 × 10^5 cells/mL in medium containing increasing concentrations of the respective antibiotic in a 96-well microtiter plate, and incubated for 20 h without shaking at 37 °C. MIC was defined as the lowest antibiotic concentration at which no visible bacterial growth was observed, corresponding to OD600 < 0.1 as measured with the Varioskan Lux Multimode Microplate Reader. For each strain 3 biological replicates were routinely used and experiment was repeated twice, with the same results.

## Electronic Supplementary Material

Below is the link to the electronic supplementary material.

Supplementary Material 1

Supplementary Material 2

## Data Availability

All data generated or analysed during this study are included in this article and its Supplementary Information files.
