# Supplementary material for: DnaA binding to oriCII regulates transcription from Pseudomonas aeruginosa gidAB-parAB locus linked to the chromosome segregation and streptomycin resistance
Source: Sci Rep. 2026 May 19;16:24067. doi: 10.1038/s41598-026-52016-6 (PMC13438810; doi:10.1038/s41598-026-52016-6)
Supplement: Supplementary file 1 [file 41598_2026_52016_MOESM2_ESM.pdf]

Supplementary Materials

DnaA binding to *oriCII* regulates transcription from *Pseudomonas aeruginosa gidAB-parAB* locus linked to the chromosome segregation and streptomycin resistance

Pawel Wawrzyniak[a,b,*], Adam Kawalek[a], Aneta Szulc[c], Monika Glinkowska[c], Grazyna Jagura-Burdzy[a], Aneta Agnieszka Bartosik[a,*]

[a] Institute of Biochemistry and Biophysics, Polish Academy of Sciences, Warsaw, Poland
[b] Department of Bacterial Genetics, Institute of Microbiology, Faculty of Biology, University of Warsaw, Warsaw, Poland
[c] Department of Bacterial Molecular Genetics, Faculty of Biology, University of Gdansk

*Address correspondence to: P. Wawrzyniak (pwawrzyniak@uw.edu.pl), A.A. Bartosik (anetab2@ibb.waw.pl)

6379091 bp
V
GCTGGAAAGCGGCAACGACTGAGCCTTTCCCACGCCTTTCCATCTCCTTCTTCCTACCTGGCTGCGGTCCTGCCACGGCCCGG
► ΔP*gidA*
DnaA box6 6-mer DnaA box7
ATACGCTCGCCTGAAACCTGTGGAAAACCCCAGGGACAGGAGGGTAGATAAACCCGCCGAAGCTCTGTGCATAACTCCACATG
► Δbox6–10
DnaA box8 DnaA box9
TTGATAACGAGCCATTCCATCCACAGCTAAAAAACGCTTATCAACAGGGCCCAGATAGGGTTTGGGACAGCCTTATCATTCGC
DnaA box10 13-mer 6-mer 6-mer
TACAGGCTATGTCTTTACTGGGCTCGACGTACTTATCAACAGAAAAGCCGCTGCTAAGTAATAAACACAAGTTCTAAAAAGAT
Δbox6–10 ◄
13-mer
ATAAACATTCCTTTCCTTTTATTTTTTGTAAGCCCGCCGAGCCGTTTTGCTCCCTCGATCTGGATTGGCTATTTTTTGTCCAG
+1 p1
GAGTCTTCATTCAGGTTCGCTAAATCCTTATACTGTCCGGCTTTCCTGAATTCCTGCTTCTGAACAGGCCGAGGTGCGTGGTG
-35 -10 ΔP*gidA* ◄
#21 →
GATTTCCCTACCCGTTTTGACGTAATCGTGATCGGCGGTGGCCATGCAGGCACCGAAGCCGCACTCGCGGCTGCGCGCATGGG
DnaA box11 DnaA box12 +1 p2
TGTGAAAACCCTGCTGTTGACCCACAATGTGGAAACCCTGGGGCAGATGAGCTGCAACCCCGCCATCGGCGGCATCGGCAAGA
GCCATCTGGTCAAGGAAATCGACGCCCTTGGCGGCGCCATGGCCGAAGCCACCGACAAGGGCGGTATCCAGTTCCGCATCCTT
← #22
AACAGTCGCAAGGGGCCGGCAGTGCGCGCCACTCGTGCACAAGCCGATCGCGTGCTCTATAAGGCCGCTATCCGCCATACACT
#35 →
GGAAAACCAGCCGAACCTGTGGATATTCCAGCAGGCCTGCGACGACCTGATCGTCGAGCAGGATCAGGTCCGCGGCGTAGTGA
DnaA box13 ← #36
CCCAGATGGGACTGCGCTTCCATGCCGACAACGTGGTCCTGACTACCGGCACCTTCCTCGGCGGACTTATCCACATCGGCCTG
GAGAACTACTCAGGCGGACGCGCCGGCGACCCGCCGTCCATCGCACTGGCGCGTCGTCTGCGTGAATTGCCGCTGCGGGTCGG
CCGGCTGAAGACCGGTACGCCGCCGCGGATCGACGGGCGCAGCGTGGACTTCTCGGTGATGACCGAGCAGCCGGGCGACACGC
CGATCCCGGTGATGTCCTTCCTCGGTTCGAAGGAACAGCATCCCGAGCAGGTCAGTTGCTGGATCACCCATACCAACGCCCGG
ACCCACGAGATCATCGCCGCCAATCTCGATCGTTCGCCGATGTATTCCGGTGTGATCGAAGGCATCGGCCCGCGCTACTGCCC
TTCGATCGAAGACAAGATCCATCGCTTCGCCGATAAGGAAAGCCATCAGGTCTTCCTCGAGCCGGAAGGCCTGACGACCCACG
AGCTGTATCCCAACGGCATTTCCACGTCCCTGCCGTTCGACGTGCAACTGCAGATCGTGCGTTCCATCCGTGGCATGGAAAAC
GCCCACATCGTGCGGCCGGGCTATGCGATCGAGTACGACTTCTTCGATCCCCGCGATCTCAGGTACAGCCTGGAGACCAAGGT
CATCGGCGGCCTGTTCTTCGCCGGCCAGATCAACGGCACCACCGGCTACGAGGAAGCTGGCGCCCAGGGCCTGCTGGCCGGCG
CCAATGCTGCGTTGCGTTCGCAAGGCAAGGACAGCTGGTGCCCGCGCCGCGACGAGGCCTACATCGGCGTGCTGGTGGACGAC
CTGATCACGCTCGGCACCCAGGAGCCGTACCGCATGTTCACCTCGCGGGCGGAATACCGCCTGATCCTCCGCGAGGACAACGC
CGACCTGCGTCTCACCGAGAAGGGACGCGAGCTGGGCCTTGTCGACGATCGGCGCTGGGCTGCCTTCGAAGCCAAGCGCGAAG
GTATCGAACGCGAGGAACAGCGGCTGAAGAGCACCTGGGTGCGGCCGAATACCCCGCAGGGCGACGCCATCGCCGAGCGCTTC
GGTACGCCGCTGACCCATGAATACAATCTGCTCAACCTGCTGAGCCGCCCCGAGATCGATTACGCCGGCCTGGTGGAGATCAC
DnaA box14
CGGCAACGCTGTGGATAACCCCCAGGTGGCCGAGCAGGTGGAGATTCGCACCAAGTACGCCGGCTACATCGACCGCCAGCAGG
AGGAAATCTCCCGTCTGCGCGCCAGCGAAGATACCCGCCTGCCTGTGGATATCGATTACCTGGGGATTTCCGGCCTGTCGAAG
GAAATCCAGAACAAGCTGAACCAGGCCCGTCCGGAAACCCTCGGACAGGCTTCGCGGATCCCCGGCGTCACGCCGGCTGCGAT
TTCGCTGTTGTTGATCCACCTGAAGAAGCGTGCCTCTGGCCGCCAACTGGAGCAGAGCGCTTGAATGTCTGCGGTAACCCAACA
► Δ*gidB'*
► Δ*gidB*
CCACGCCGACGAACTCGCGCGCGGTGCTGACGAGCTAGGCGTCGCCCTCGACGCCGACAAGCAACGCCAGCTGCTGGCCTACC
TGGCCCTGCTGATCAAATGGAACAAGGCCTACAACCTGACCGCCGTACGCGACCCGGACGAGATGGTTTCGCGCCACCTGCTC
#37 →
GACAGCCTGAGCATCGTGCCTTATGCCGAGGCTGGGGATAACTGGCTGGATGTCGGCAGCGGCGGCGGCATGCCCGGCGTGCC
GCTGGCCATTCTGTTCCCGGAAAAGCGCCTGACCCTGCTCGACAGCAACGGCAAGAAGACCCGCTTCCTGACCCAGGTGAAGC
← #38
TCGAACTGAAGCTGGCCAACCTCGAAGTCGTGCACAGCCGGGTGGAAGCCTTCCGTCCCGAGAGCCCCTTCGATGGCATCGTT
TCCCGCGCTTTCAGCAGCCTGGAGGACTTCGCCAACTGGACCCGCCACCTCGGTGGCCAGGAAACCCGCTGGCTGGCGATGAA
#23 → +1 p3
GGGCGTGCATCCGAACGAAGAGCTGGCGGCACTCCCGGAGGATTTCCGGGTCGAAGCCGAGCACGCGCTGGCGGTACCCGGTT
Δ*gidB'*◄
+1 p4
GCCAAGGTCAGCGGCATCTGCTGATACTGCGGCGCACAGCATGAGGGGGAACAGGGATAACCATGGCTAAGGTATTCGCGATC
Δ*gidB* ◄
GCCAACCAGAAAGGCGGTGTCGGCAAGACCACGACCTGTATCAACCTCGCGGCCTCCCTCGTGGCGACCAAGCGCCGCGTCCT
CCTGCTGGACCT*GGATCC*CCAGGGCAACGCCACCACCGGCAGCGGCATCGACAAGCACAACCTGGAGCACTCCATCTACGACG
TGCTCACCGGCGAGTGCAACCTCGCCGAGGCCATGCAGTTCTCCGAACACGGCGGCTACCAGTTGCTGCCGGCCAACCGCGAC

```
                            ← #24
CTGACCGCCGCGGAAGTGGTGCTGCTGGAAATGGACATGAAGGAAAATCGCCTGCGCAACGCGCTGGCGCCGATCCGCGAGAA
CTACGACTACATCCTCATCGACTGCCCGCCGTCGCTGTCGATGCTCACCGTCAACGCGCTGACCGCCGCCGACGGCGTGATCA
                                                        #39 →
TCCCCATGCAGTGCGAGTACTACGCGCTGGAGGGGCTGACCGACCTGATGAACAGCATCCAGCGCATCGGCCAGTTGCTCAAC
                                          ← #40
CCGACGCTGAAGATCGAGGGTCTGTTGCGGACCATGTACGATCCGCGCATCAGCCTGACCAACGATGTCTCCGCGCAGTTGCA
GGAGCATTTCGGCGACACCCTCTACAGCACCGTCATCCCGCGTAACGTGCGCCTCGCCGAAGCCCCCAGCTTCGGCATGCCGG
CGCTGGTTTACGACAAGCAATCCCGCGGCGCCATCGCCTACCTGGCGCTGGCCGGCGAGCTGGTCCGCCGCCAGCGCGCCAAA
GGCCGCGCCGCAACCGCATAAGGAACCCGCATGGCAGCCAAGAAACGTGGACTGGGACGCGGGCTCGATGCCCTGCTGGGCGG
      #25 →
CTCGAGTCCCGCCAAACTGCAGGAGGAAGCCGTACAGGCCGACCGCCGCGAGTTGCAGCAGATCCCCCTCGACCTGATGCAGC
GCGGCAAGTACCAGCCGCGTCGCGACATGGACCCTCAGGCGCTGGAGGAACTGGCGCAGTCGATCAAGGCCCAGGGCGTGATG
                        ← #26
CAGCCCATCGTGGTGCGGCCGATCGCCAATGGCCGCTACGAGATCATTGCCGGCGAGCGGCGCTGGCGGGCCAGCCAGCAGGC
CGGACTGGAAAAGATCCCGGCGCTGGTGCGCGACGTGCCGGACGAAGCGGCCATCGCCATGGCGCTGATCGAGAACATCCAGC
GCGAGGACCTCAACCCGATCGAGGAAGCCGCTGCCCTGCAGCGCTTGCAGCAGGAGTTCCAGCTGACCCAGCAGCAGGTCGCG
                                                                              ← #27
GAAGCGGTCGGCAAGTCGCGCGCGACCATTACCAACCTGCTGCGCCTGATCGGCCTGCCGGAAGAGATCAAGACCCTGCTTTC
CCATGGCGACCTGGAGATGGGCCATGCCCGTGCGCTCCTCGGTCTGCCGGCAGAACGGCAGGTGGAAGGGGCGCGACATGTTG
TCGCACATGGGCTGACCGTACGTCAGACCGAAGCCCTGGTTCGGCAGTGGACGCATGCCCCTGGCAAGCCTGCGGGACCGGTC
                                                                               #41 →
AAGAGCGACCCGGACATCGCCCGCCTGGAACAGCGCCTGGCCGAGCGCCTGGGCGCTTCGGTGCAGATCCGGCATGGTCAGAA
                                                                     ← #42
GGGCAAGGGACAGTTGGTCATCCGCTACAGCTCCCTGGATGAACTGCAGGGCGTTCTCGCCCACATCCGTTGACACACTTTGG
GTTGTAGCGGGTAGTCGGAAATCACTACCAGCCAGTTGAACAGGGGGGACTCCCCCCCTATACTCTGCGCGCTATTTTGTGGG
CACTTTTTATGCCAAGTCGTTGATTTGGCAGTGCCGACACCAGAAGACTGAGAGCGCGATGGAGTCCCGCATGCCAAGTCGCC
                          ← #28
TGCCGGCATTCAGATTGCTGCTCGTCCAACTGG
                                  ^
                              6374052 bp
```

gidA **–** ***gidB*** **–** ***parA*** **–** ***parB***
+1 – 5′ ends of transcripts (from dRNA-SEQ)
NNNNNN **– DNA fragment cloned in pCMp1box**
NNN **– DNA fragment cloned in pCMbox**
NNN **– DNA fragment cloned in pCMp1**
NNN **– DNA fragment cloned in pCMp2**
NNN **– DNA fragment cloned in pCMp3**
NNN **– DNA fragment cloned in pCMp4**
NNN – RT-PCR and qRT-PCR DNA primers
► ◄ – start and end of deletion regions
NNN – 6-mer-like sequences
NNN – 13-mer sequences
NNN – DnaA box

FIGURE S1

DNA sequence of *gidAB-parAB* operon, figure legend is at the bottom of the graphic.

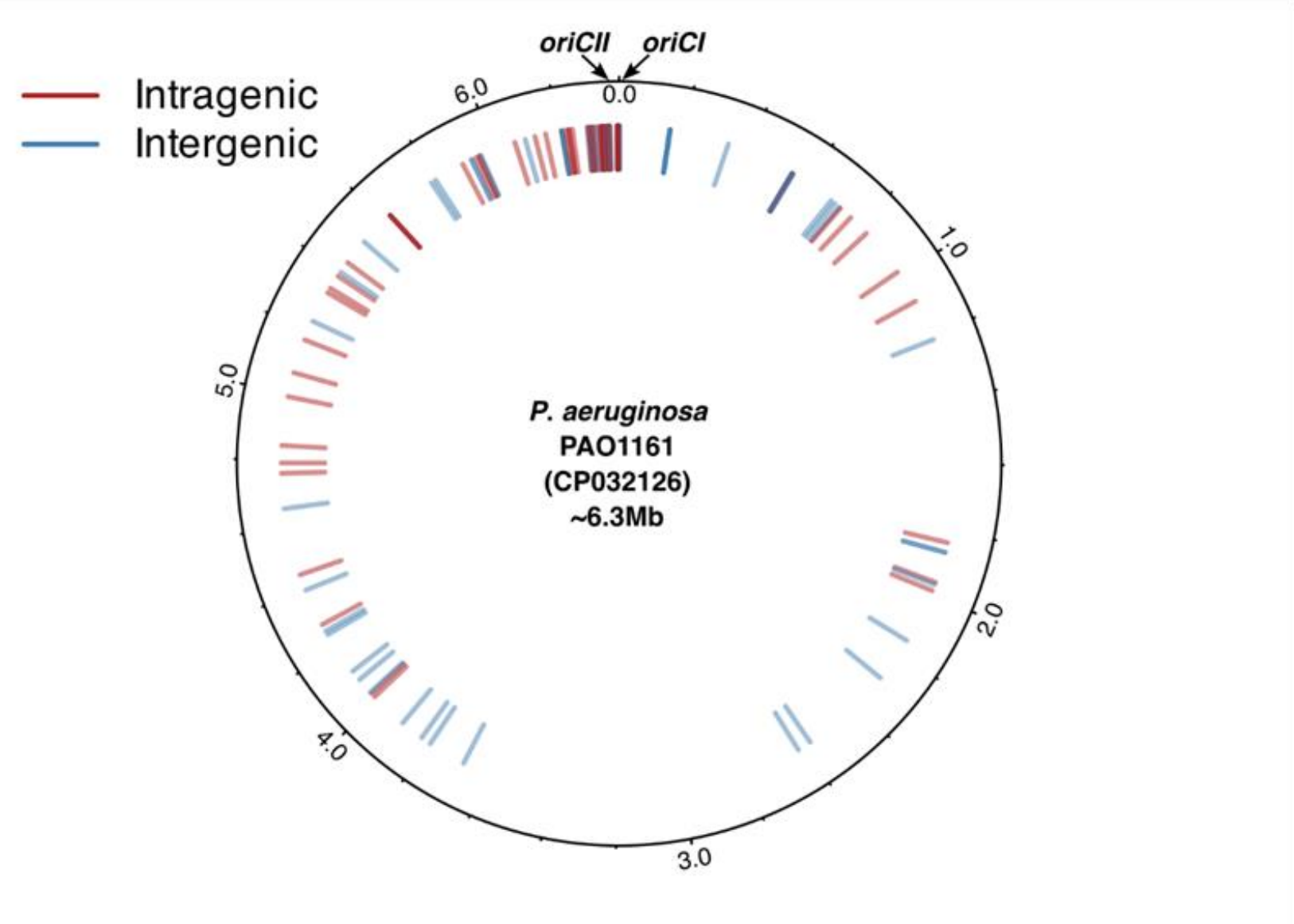

FIGURE S2

Distribution of DnaA box sequences (consensus: TTWTNCACA) in the *P. aeruginosa* (PAO1161, Acc. No. NZ_CP032126.1) chromosome. Boxes located within open reading frames are marked in red. Boxes located in intergenic regions are marked in blue.

DnaA protein overexpression and purification

P. aeruginosa DnaA protein was overexpressed from pET28-MBP-*dnaA* construct, in fusion with a His6-MBP-TEV tandem tag, consisting of hexahistidine peptide, maltose binding protein (MBP) sequence and TEV protease cleavage site. MBP tag was employed to increase *P. aeruginosa* DnaA solubility. *E. coli* BL21(DE3) Δ*clpP* strain (Table S3) was used as a host, to reduce DnaA proteolysis.

The host strain bearing the pET28-MBP-dnaA plasmid was grown at 30°C in 1.5 L of LB medium with aeration. At OD600 ~ 0.8-1, DnaA overproduction was induced with 200 µM IPTG and the culture was further incubated with shaking for 5 h at 26°C. Subsequently, bacterial cells were harvested by centrifugation; the pellet was flash-frozen and stored at -80°C. For DnaA purification the cell pellet was resuspended in 40 ml of Lysis buffer (50 mM HEPES pH 7.5, 200 mM K-glutamate, 15% sucrose, 15 mM imidazole, 0.2% TritonX, 0.5 mM TCEP, 1mM PMSF). Lysozyme was added to the final concentration of 0.5 mg/ml and the sample was incubated at 30 min on ice, followed by cell disruption by sonication. Next , 2 µl of viscolase (A&A Biotechnology) was added after sonication to reduce sample viscosity and incubation was carried out on ice for another 30 min. The lysate was cleared by centrifugation at 48000 x g for 1h. In the first stage of purification, the lysate was filtered and loaded on the HisTrap HP column (5 ml, GE Healthcare) pre-equilibrated with the Lysis buffer devoid of TritonX, TCEP and PMSF. The column was washed with 2 volumes of A1 buffer (50 mM HEPES pH 7.2, 200 mM K-glutamate, 200 mM KCl, 10% glycerol, 20 mM sucrose, 20 mM imidazole, 10 mM Mg-acetate, 0.5 mM TCEP), 5 volumes of IEX2 buffer (50 mM HEPES pH 7.2, 1M K-glutamate, 10% glycerol, 25 mM sucrose, 10 mM Mg-acetate, 0.5 mM TCEP) and then with a step gradient of B1/A buffer (5%-4ml, 10%-4ml, 15%-15ml, 20%-10 ml) (B1: 50 mM HEPES pH 7.2, 200 mM K-glutamate, 200 mM KCl, 10% glycerol, 400 mM imidazole, 20 mM sucrose, 5 mM Mg-acetate, 0.5 mM TCEP). DnaA elution was carried out with 50 ml of linear B1/A1 gradient (20-100% B1/A1). 1 ml fractions containing the DnaA protein were pooled and dialysed overnight at 4°C to remove imidazole, in a buffer containing 50 mM HEPES pH 7.2, 500 mM K-glutamate, 20% sucrose, 10 mM Mg-acetate, 0.5 mM TCEP. Next day, heparin column HiTrap Heparin HP (5ml, GE Healthcare) was equilibrated with IEX1 buffer (50 mM HEPES pH 7.2, 100 mM K-glutamate, 10% glycerol, 25 mM sucrose, 10 mM Mg-acetate, 0.5 mM TCEP). The dialysed DnaA protein was diluted 5-6 times in A0 buffer (50 mM HEPES pH 7.2, 10% glycerol, 20 mM sucrose, 10 mM Mg-acetate) until conductivity was lower than that of IEX1 buffer. After sample loading, the column was washed

with 25 ml of IEX1 buffer and then elution was carried out with a linear gradient of IEX2/IEX1 buffers (50 ml). 1 ml fractions containing DnaA were pooled, supplemented with TEV protease (25 mg/ml final conc.; purified in-house as previously described [Walter et al., 2020]), 0.5 mM EDTA and 1 mM DTT, and subsequently dialysed overnight as described above. His_6-MBP tag was removed on HisTrap HP column, as described above and the DnaA protein was collected in the flow-through, concentrated and stored in the dialysis buffer (50 mM HEPES pH 7.2, 500 mM K-glutamate, 20% sucrose, 10 mM Mg-acetate, 0.5 mM TCEP) (Supplementary Fig. S3).

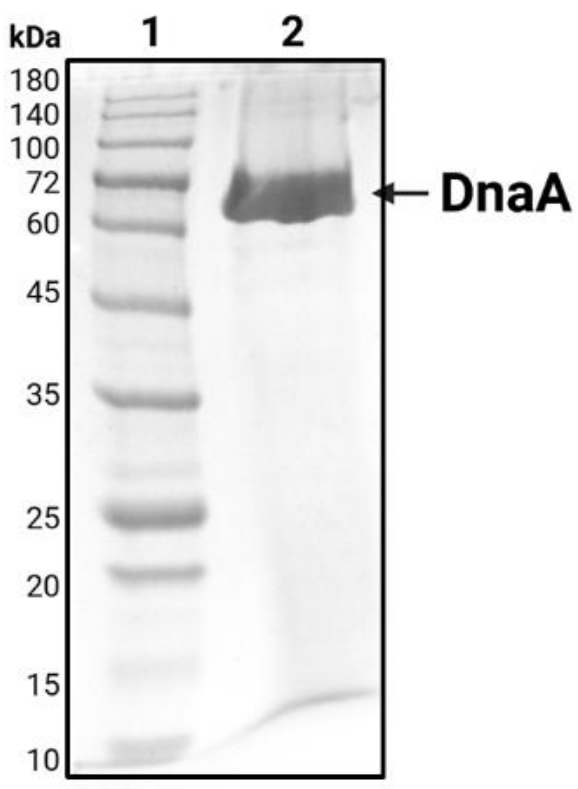

FIGURE S3

SDS-PAGE analysis of purified *P. aeruginosa* DnaA protein. Wells from left to right: 1: molecular weight marker (JustBlue Protein Marker, Cat. No. MWP05); 2: final purified DnaA protein (50 µg) after the three-step purification procedure, showing a single band at the expected molecular mass.

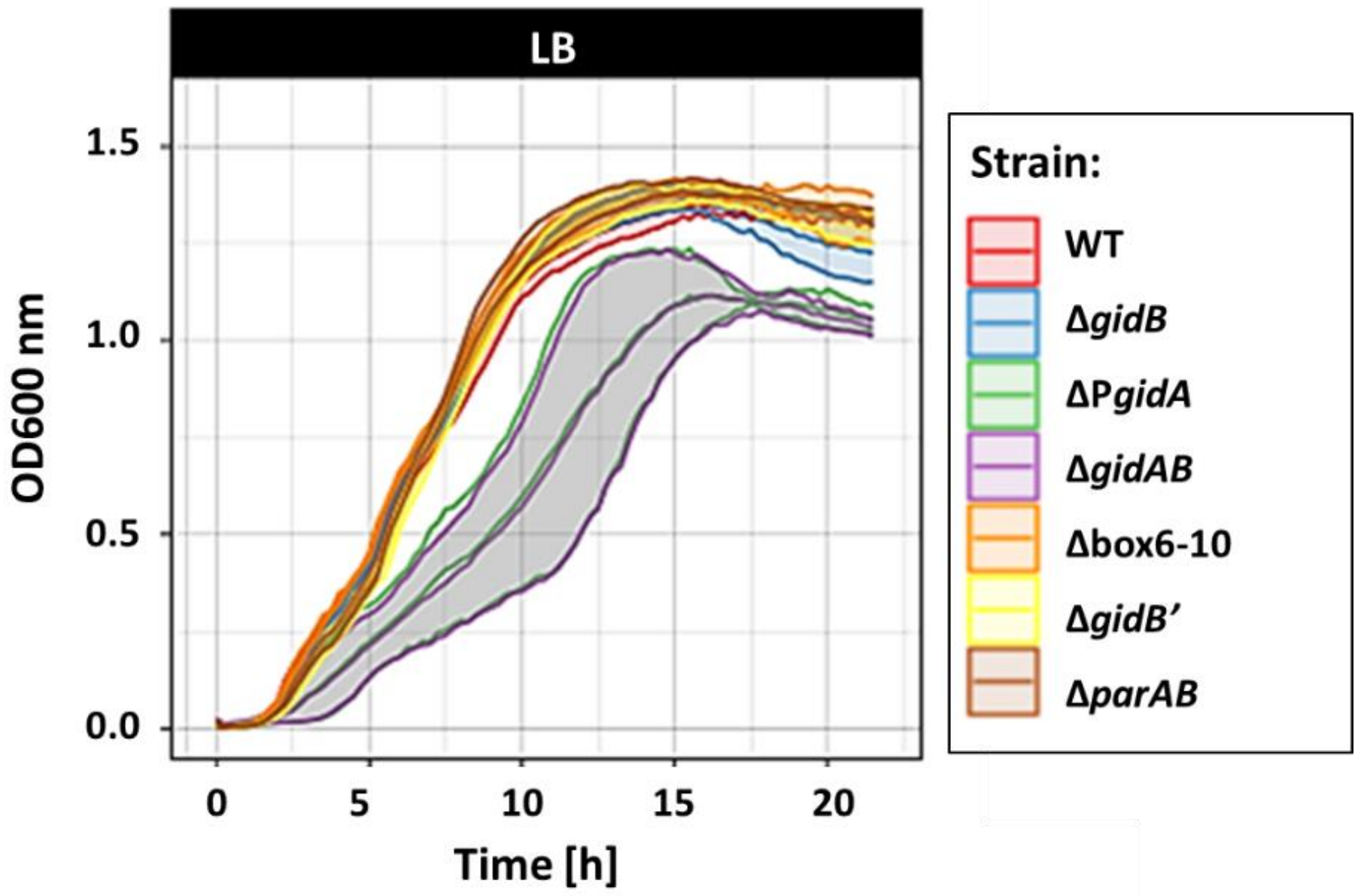

FIGURE S4
Growth of WT PAO1161 and its mutant derivatives in LB medium at 37°C with shaking. Growth kinetics were monitored by measurements of optical density at 600 nm (OD600) in 96-well plates at 37 °C using a Varioskan Lux Multimode Microplate Reader and SkanIt RE 7.0.2.5 software (Thermo Fisher Scientific, Waltham, MA, USA). Data are presented as the mean ±SD of 2 biological replicates, each performed with 2 technical replicates.

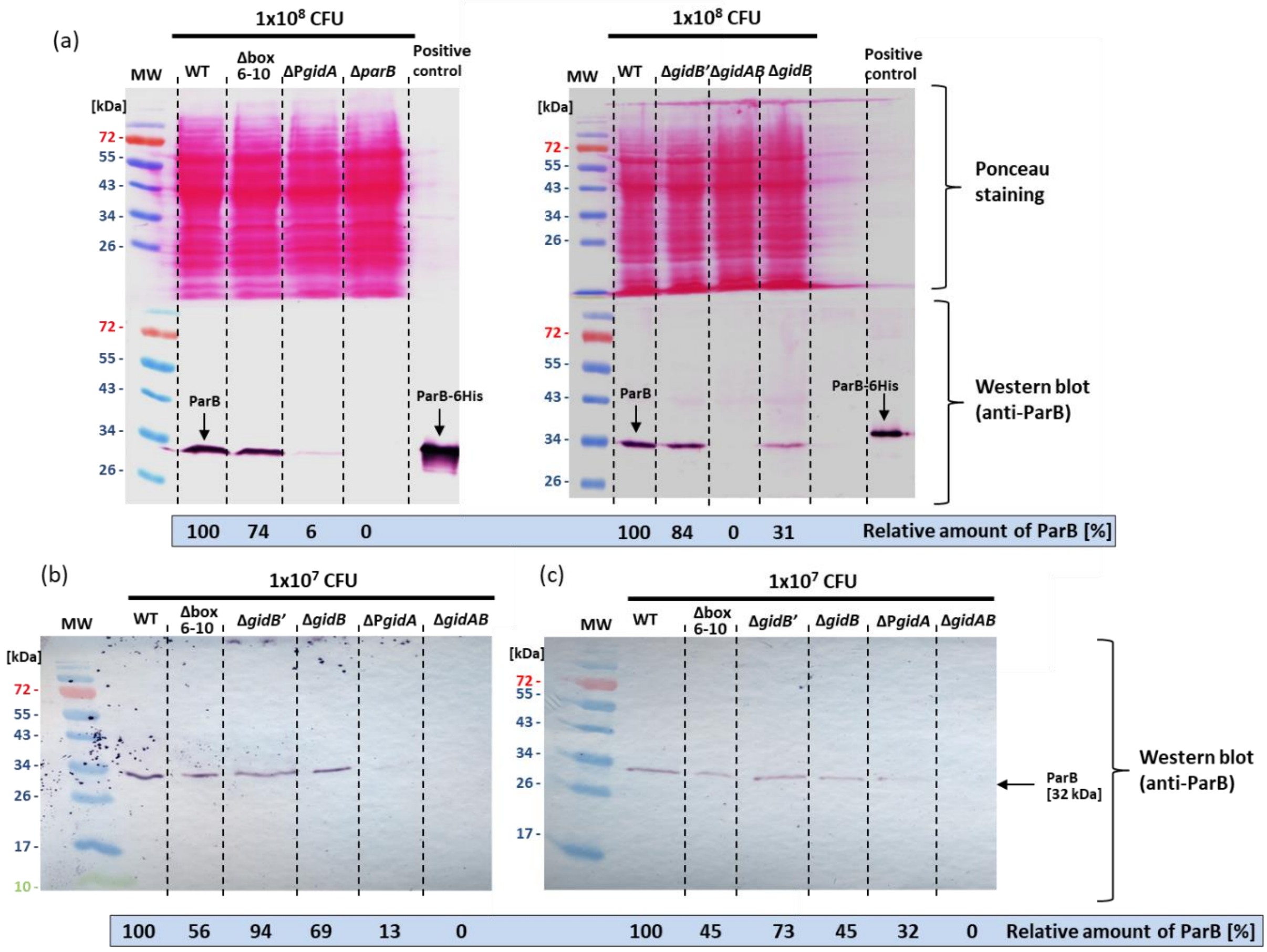

FIGURE S5
Densitometric analysis of relative ParB levels. Western blot of endogenous ParB in PAO1161 and its deletion mutants during the exponential growth phase. Culture samples were harvested by centrifugation, resuspended in 1x SDS-PAGE loading buffer, and boiled. Samples were normalized to equivalent CFU counts and separated via 12% SDS-PAGE (see Fig. 4d in the main text). Proteins were transferred onto nitrocellulose membranes and detected with anti-ParB antibodies. Band intensities were quantified densitometrically using ImageJ software. Results from three independent replicates are presented (panels a–c).

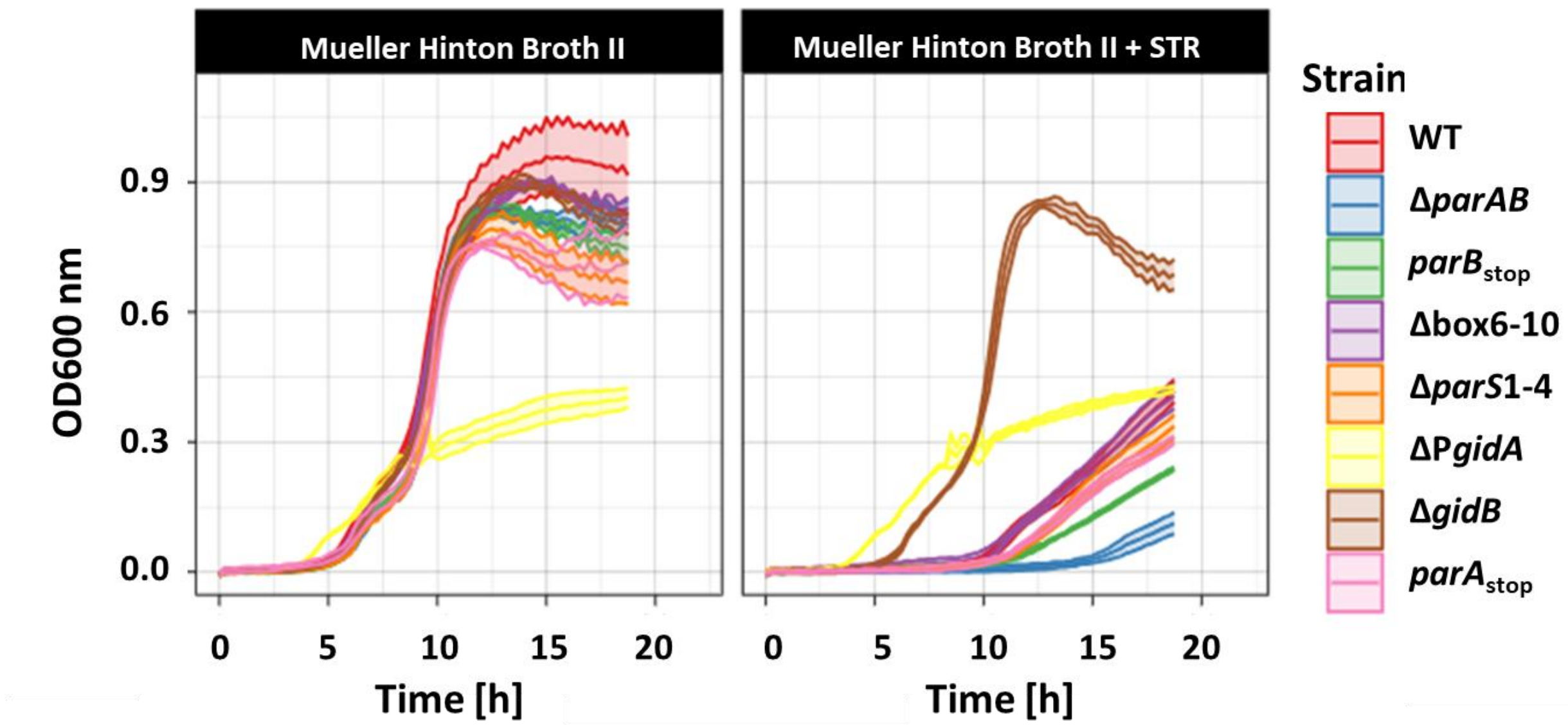

FIGURE S6
Growth of WT PAO1161 and its mutant derivatives was assessed in MH Broth II medium or in MH Broth II medium with 6 µg/ml streptomycin at 37°C with shaking. Growth kinetics were monitored by measurements of optical density at 600 nm (OD600) in 96-well plates at 37 °C using a Varioskan Lux Multimode Microplate Reader and SkanIt RE 7.0.2.5 software (Thermo Fisher Scientific, Waltham, MA, USA). Data are presented as the mean ±SD of 2 biological replicates, each performed with 2 technical replicates.

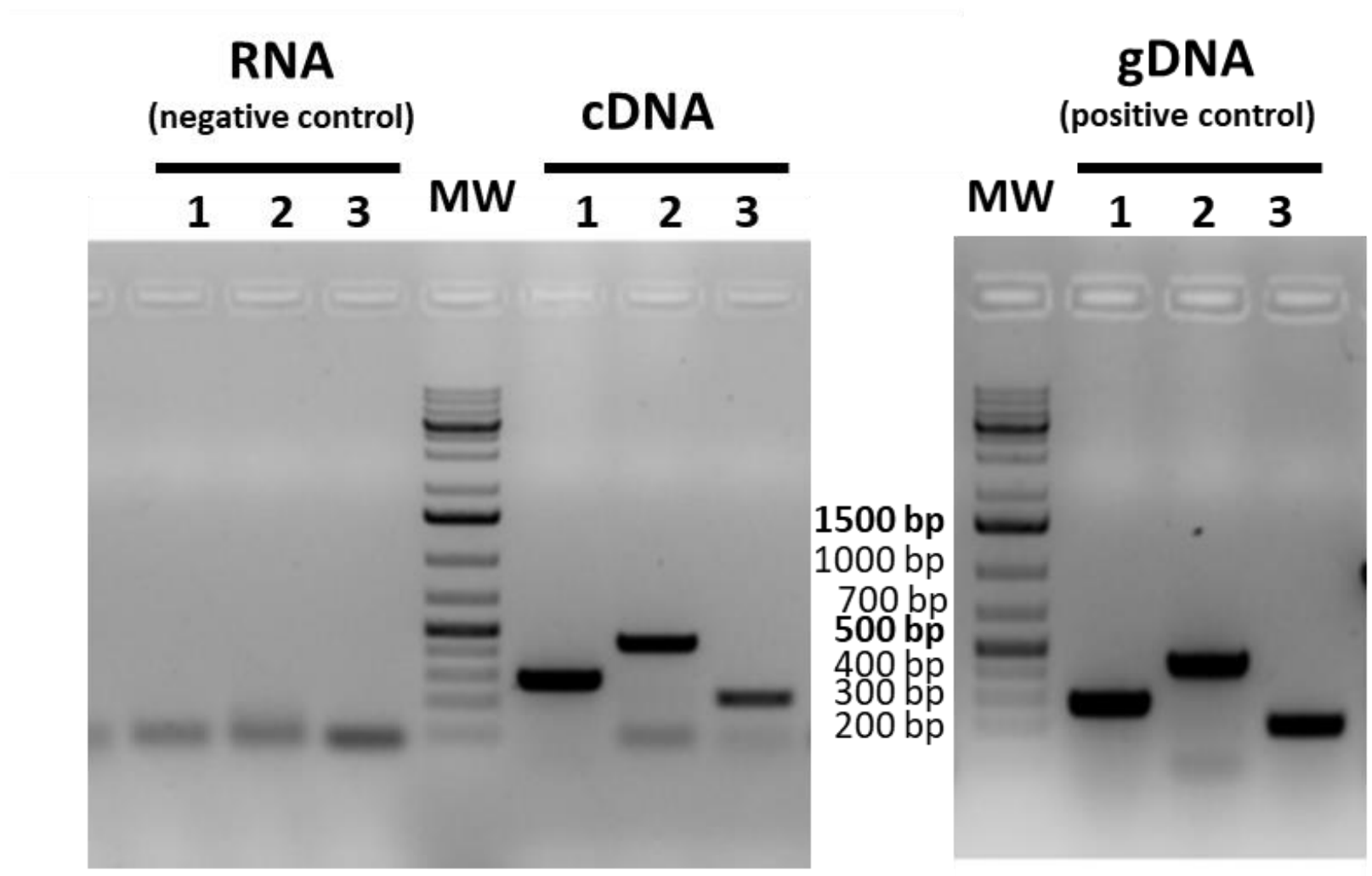

FIGURE S7
Original gels: DNA electrophoresis of RT-PCR products (related to Fig. 1d in the main text).

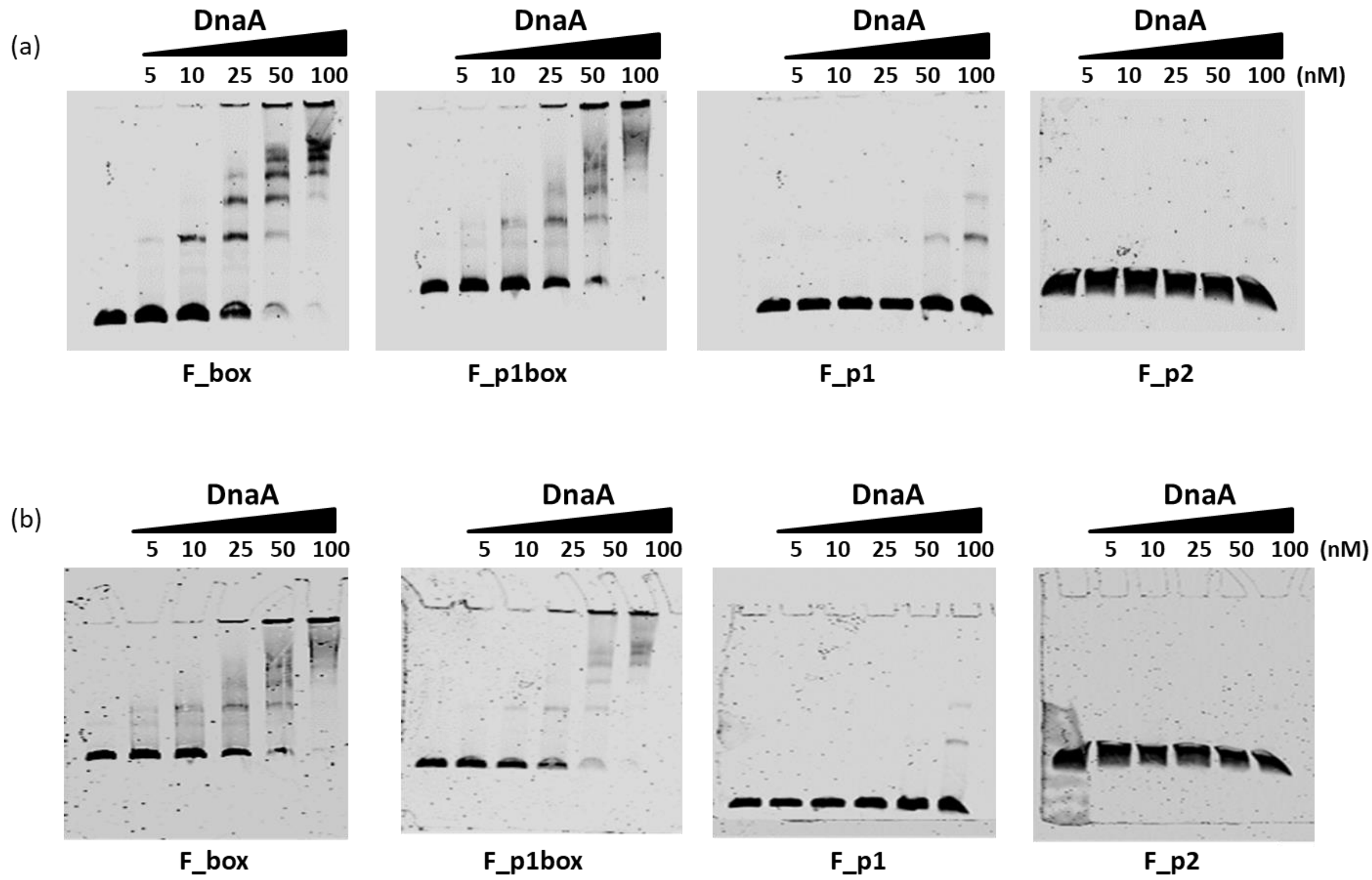

FIGURE S8
Original gels: electrophoretic mobility shift assay (EMSA), results from two independent replicates are shown (panels (a) – related to Fig. 3 in the main text, and (b) – second independent experiment).

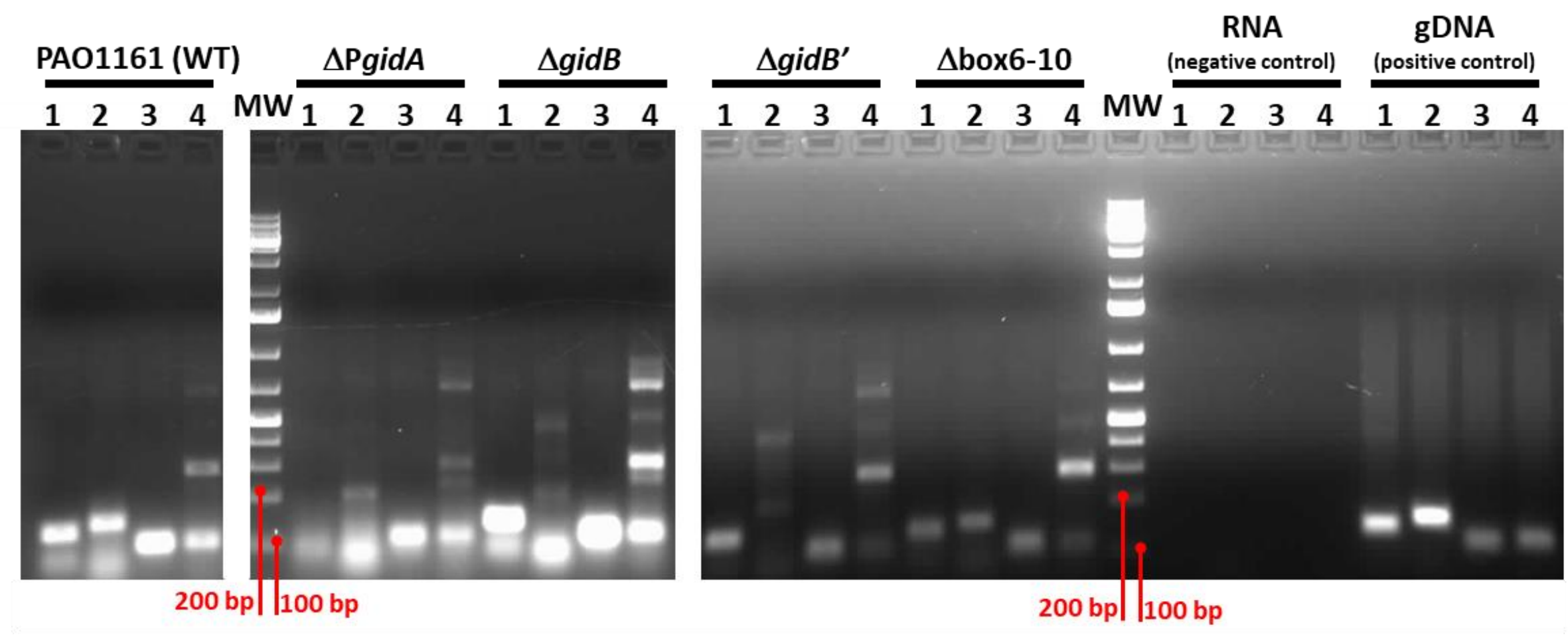

FIGURE S9
Original gels: DNA electrophoresis of RT-PCR products (related to Fig. 4c in the main text).

Table S2. Bacterial plasmids used in this study

Plasmid	Description	
**pAKE600**	suicide vector, *oriV*$_{MB1}$, *sacB* gene, Ap^R	[El-Sayed et al., 2001]
**pAKE600P*gidA***	Two DNA fragments amplified on PAO1161 chromosome with the use of primers #1#2and #3#4 were cloned into pAKE600 between EcoRI and BamHI restriction sites to delete PA5565 *gidA**(*mnmG*)** (D3C65_RS30235)** promoter, deletion coordinates (6378598-6379008)**	this study
**pAKE600*gidB***	Two DNA fragments amplified on PAO1161 chromosome with the use of primers #5#6 and #7#8 were cloned into pAKE600 between EcoRI and HindIII restriction sites to delete PA5564 *gidB**(*rsmG*)** (D3C65_RS30210)**, deletion coordinates (6376068-6376701)**	this study
**pAKE600*gidAB***	Two DNA fragments amplified on PAO1161 chromosome with the use of primers #1#2 and #7#8 were cloned into pAKE600 between EcoRI and HindIII restriction sites to delete PA5556 *gidA**(*mnmG*)** promoter, *gidA**(*mnmG*)** and PA5564 *gidB**(*rsmG*)** (D3C65_RS30235, D3C65_RS30210)**, deletion coordinates (6376068-6379008)**	this study
**pAKE600*gidB'***	Two DNA fragments amplified on PAO1161 chromosome with the use of primers #5#6 and #8#9 were cloned into pAKE600 between EcoRI and HindIII restriction sites to delete *5'*-end of *gidB**(*rsmG*)** (D3C65_RS30210)**, deletion coordinates (6376114-6376701)**	this study
**pAKE600box6-10**	Two DNA fragments amplified on PAO1161 chromosome with the use of primers #4#12 and #10#11 were cloned into pAKE600 between EcoRI and BamHI restriction sites to delete five DnaA boxes of *oriCII,* deletion coordinates (6378804-6378993del)**	this study
**pCM132**	Dual replicon, promoter-probe vector, *oriV*$_{MB1}$, *oriV*$_{RK2}$, *oriT*, *traJ'*, *trfA*, Km^R, promoter-less *lacZ*	[Marx and Lidstrom, 2001]
**pCMp1box**	DNA fragment containing *gidA** (D3C65_RS30235)** promoter with DnaA boxes 6-10, coordinates (6378583-6379008), amplified on PAO1161 chromosome with the use of primers #13#14 was cloned into pCM132 between EcoRI and BglII restriction sites	this study
**pCMbox**	DNA fragment containing DnaA boxes 6-10, coordinates (6378775-6379008)**, amplified on PAO1161 chromosome with the use of primers #11#15 was cloned into pCM132 between EcoRI and BglII restriction sites	this study
**pCMp1**	DNA fragment containing *gidA** (D3C65_RS30235)** promoter, coordinates (6378583-6378774)**, amplified on PAO1161 chromosome with the use of primers #15#16 was cloned into pCM132 between EcoRI and BglII restriction sites	this study
**pCMp2**	DNA fragment containing *5'*-end of *gidA** (D3C65_RS30235)**, coordinates (6378470-6378582)**, amplified on PAO1161 chromosome with the use of primers #17#18 was cloned into pCM132 between EcoRI and BglII restriction sites	this study
**pCMp3**	DNA fragment containing part of *gidB** (D3C65_RS30210)**, amplified on PAO1161 chromosome with the use of primers #19#20 was digested using native KpnI site and DNA fragment, coordinates (6376115-6376512)** was cloned into pCM132 between KpnI and BglII restriction sites	this study
**pCMp4**	DNA fragment containing 3'-end of *gidB** (D3C65_RS30210)** and intergenic region *gidB**-*parA* (D3C65_RS30205)**, amplified on PAO1161 chromosome with the use of primers #19#20 was digested using native KpnI site and DNA fragment, coordinates (6376042-6376114)** was cloned into pCM132 between KpnI and EcoRI restriction sites	this study
**pKGB8**	Cm^R; IncA/C broad-host-range expression vector, *mob*, *araC*-p*BAD*	[Kawalek et al., 2017]
**pKGB9**	pKGB8 with cloned *parB* gene from PAO1161	[Kawalek et al., 2017]
**pBAD24**	*araC*-p*BAD* expression vector, *oriV*$_{MB1}$, Ap^R	Addgene
**pMKB4**	pAKE600 derivative carrying PAO1161 *parA*-*parB* genes	[Kusiak et al., 2022]
**pAKE600*parB*stop**	pMKB4 derivative modified by the PCR-based site directed mutagenesis using primers #49 and #50 resulting in *parB* with tandem STOP codons immediately after ATG start codon	this study
**pET28a-His6-MBP-TEV**	The ORF fragment encoding K27 to K396 of the *E. coli* MalE protein together with a TEV protease cleavage site (ENLYFQGS) was cloned using the NheI and BamHI restriction sites of the pET28 bacterial expression plasmid (Novagen)	[Currin et al., 2015]
**pET28-MBP-dnaA**	DNA fragment containing PAO1/PAO1161 *dnaA* gene sequence was PCR amplified with the use of primers #43#44 and inserted into pET28a-His6-MBP-TEV vector downstream of the sequence encoding TEV cleavage site via restriction-free cloning method, as described in van den Ent and Löwe (2006)	this study
**pUC-p1box**	DNA fragment containing *gidA** (D3C65_RS30235)** promoter with DnaA boxes 6-10, coordinates (6378583-6379008), amplified on PAO1161 chromosome with the use of primers #13#14 was cloned into pUC18 between EcoRI and BamHI restriction sites	this study
**pUC-p1**	DNA fragment containing *gidA** (D3C65_RS30235)** promoter, coordinates (6378583-6378774)**, amplified on PAO1161 chromosome with the use of primers #15#16 was cloned into pUC18 between EcoRI and BamHI restriction sites	this study
**pUC-box**	DNA fragment containing DnaA boxes 6-10, coordinates (6378775-6379008)**, amplified on PAO1161 chromosome with the use of primers #13#15 was cloned into pUC18 between EcoRI and BamHI restriction sites	this study
**pUC-p2**	DNA fragment containing *5'*-end of *gidA** (D3C65_RS30235)**, coordinates (6378470-6378582)**, amplified on PAO1161 chromosome with the use of primers #17#18 was cloned into pUC18 between EcoRI and BamHI restriction sites	this study
**pKAB240**	pBAD24 with NsiI restriction site between T$_{rrnB}$ and *blap*	[Kawalek et al., 2017]
**pKAB603**	pAKE600 with *P. aeruginosa* genomic fragment coordinates (6090756-6092192)** with PstI at position 6091513** nt and deletion of EcoRI restriction site	[Kawalek et al., 2017]
**pAKW882**	PA0001 (*dnaA*) was amplified using primers #47 and #48, digested with XbaI/SalI and cloned into pUC19 yielding plasmid pAKW816. The gene was excised as MunI/SalI fragment and combined with AraC araBADp promoter by ligation with a pBAD24 derivative	this study

	pKAB240, digested with EcoRI/SalI. The combined cassette was excised using NsiI and ligated with PstI digested pKAB603, yielding plasmid pAKW882, allowing integration of this cassette in the intergenic region between PA5412 and PA5413.	

*Designation of genes in PAO1 genome

**Designation of genes and deletion coordinates in brackets according to the genome of PAO1161 (GeneBank Acc. No CP032126)

Table S3. Bacterial strains used in this study

Bacterial strain	Description	
***E. coli* S17-1**	*hsdR recA pro RP4-2 (Tc::Mu; Km::Tn7)(λpir)*	[Simon et al., 1983]
***E. coli* MC1061**	*araD139 Δ(ara- leu)7697 ΔlacX74 galU galK hsr⁻ hsm⁺ strA*	[Casadaban and Cohen, 1980]
***E. coli* BL21(DE3)**	*E. coli B dcm ompT hsdS*($r^{B-}m^{B-}$) *gal*	Invitrogen
E. coli* BL21(DE3) *ΔclpP	*E. coli* BL21(DE3) *clpP::cmr*	[Glaza et al., 2015]
***P. aeruginosa* PAO1161**	*leu r⁻ m⁻* Rif^R, (GeneBank Acc. No CP032126)	[Kawalek et al., 2020]
***parA*$_{stop}$**	PAO1161 with a stop codon in the 5' end of *parA* (D3C65_RS30205)	[Lasocki et al., 2007]
***parB*$_{stop}$**	PAO1161 modified with the use of pAKE600parB$_{stop}$ vector	this study
**Δ*parAB***	PAO1161 with deletion of both *parA* and *parB* genes	[Kawalek et al., 2023]
**Δ*parS*1-4**	PAO1161 parSmut15 with four *parS* sequences modified (here referred to as Δ*parS*1-4)	[Jecz et al., 2015]
**ΔP*gidA***	PAO1161 modified with the use of pAKE600P*gidA* vector	this study
**Δ*gidB***	PAO1161 modified with the use of pAKE600*gidB* vector	this study
**Δ*gidAB***	PAO1161 modified with the use of pAKE600*gidAB* vector	this study
**Δ*gidB'***	PAO1161 modified with the use of pAKE600*gidB'* vector	this study
**Δbox6-10**	PAO1161 modified with the use of pAKE600box6-10 vector	this study
**I1A_*dnaA***	PAO1161 modified with the use of pAKW882 vector	this study
**I1A_BADp (PAO1161::*araBAD*p)**	PAO1161 with *araC araBAD*p T_{rrnB}[1] cassette integrated in intergenic region between PA5412 and PA5413 (at position 6091513 nt)	[Kawalek et al., 2017]
**PAO1**	PAO1-CECT4122 from the Spanish Type Culture Collection	[Grudniak et al., 2018]

[1]T_{rrnB} transcriptional terminator *of E. coli rrnB* gene

Table S4. Sequences of oligonucleotides used in this study

	Oligo	Sequence 5'-3'
**1**	p5565AF	TAA**GAATTC**TGGACTCCGTCGATACCG
**2**	p5565AR	TAA**TCTAGA**AGGCTCAGTCGTTGCCGC
**3**	p5565BF	TAA**TCTAGAG**AGCTCGGTGGATTTCCCTACCCG
**4**	p5565BR	TAA**GGATCC**TCGGCTTGTGCACGAGTG
**5**	5564o1AF	TAA**TCTAGA**CATCAAGCGCTCTGCTCC
**6**	5564o1AR	TAA**GAATTC**TTCGCACCAAGTACGCCG
**7**	5564o1BF	TAA**TCTAGA**CAGCATGAGGGGGAACAG
**8**	5564o1BR	TAA**AAGCTT**GCCGTGTTCGGAGAACTG
**9**	5564o1BF2	TAA**TCTAGA**TAGTACCCGGTTGCCAAGGTC
**10**	BOXDELAF	GCT**GAATTC**GAATGCCAGGTCGGACAG
**11**	BOXDELAR	TAA**TCTAGA**TTCAGGCGAGCGTATCCG
**12**	BOXDELBF	TAA**TCTAGA**CAGAAAAGCCGCTGCTAAG
**13**	GIDAP1F	TCC**GAATTC**ATACGCTCGCCTGAAACC
**14**	GIDAP4R	TAA**GGATCC**GTAGGGAAATCCACCACGC
**15**	GIDAP3R	TAA**GGATCC**GTGTTTATTACTTAGCAGCGGC
**16**	GIDAP2F	TCC**GAATTC**GCCGCTGCTAAGTAATAAACAC
**17**	GIDAP5F	TCC**GAATTC**CCGTTTTGACGTAATCGTGATC
**18**	GIDAP6R	TAA**GGATCC**CCAGGGTTTCCACATTGTGG
**19**	GIDBP1F	TCC**GAATTC**CTGAGCATCGTGCCTTATGC
**20**	GIDBP2R	TAA**GGATCC**GGTTATCCCTGTTCCCCCTC
**21**	GIDAF1	GACGTAATCGTGATCGGCGGTG
**22**	GIDAR3	ATCGGCTTGTGCACGAGTGGC
**23**	GIDBF12	CGTGCATCCGAACGAAGAGCTG
**24**	PARAR1_2	CCATTTCCAGCAGCACCACTTCC
**25**	PARBF1	CTCGAGTCCCGCCAAACTGC
**26**	PARBR3	TCGTAGCGGCCATTGGCGATC
**27**	PARBR	GAAAGCAGGGTCTTGATCTC
**28**	PARBZ1R	CCAGTTGGACGAGCAGCAATCTG
**29**	BD0766F	TAA**GAATTC**TATGCATACCCTAAAACGCTGTATG
**30**	BD0766R	TAA**AAGCTT**TTATTCGGCCAGCTTGAAGG
**31**	0766AF	TAA**GAATTC**CTCCGTCCTCTTCTATCT
**32**	0766AR2	TTA**TCTAGA**CATCGACTACAGCTCCCG
**33**	0766BF	TAA**TCTAGA**GAATAAGCCGGCTGGCGA
**34**	0766BR2	TTA**AAGCTT**CCTCCATTTCGCGATCGG
**35**	qGIDAF	ATCGTCGAGCAGGATCAGG
**36**	qGIDAR	AGTAGTTCTCCAGGCCGATG
**37**	qGIDBF	CTGGGGATAACTGGCTGGAT

**38**	qGIDBR	GCTTCAGTTCGAGCTTCACC
**39**	parAq1F	TGACCGACCTGATGAACAGC
**40**	parAq1R	GGATCGTACATGGTCAGAA
**41**	parBq1F	CAGATCCGGCATGGTCAGAA
**42**	parBq2R	CAACGGATGTGGGCGAGAA
**43**	PA-DnaA_P_F	GCGAGAATTTGTATTTTCAGGGTCGACAAGTGTCCGTGGAACTTTGGC
**44**	PA-DnaA_P_R	GCTCGAGTGCGGCCTTGCAAGCTCAGGTTGTCAGGGTACGC
**45**	FAM-PUC1	CAGGAAACAGCTATGAC
**46**	PUC2	GTAAAACGACGGCCAGT
**47**	1014_dA46F5XF	TATA**TCTAGACAATTG**GTGTCCGTGGAACTTTGGC
**48**	1015_dA46F3SR	TATA**GTCGAC**TCAGGTTGTCAGGGTACGCA
**49**	PaBstop1	ACCGCATAAGGATCCCGCATGTGAGCCTAAGAAACGTGGAC
**50**	paBstop2	CACGTTTCTTAGGCTCACATGCGGGATCCTTATGCGGTTGC

Literature:

Bartosik AA, Lasocki K, Mierzejewska J, Thomas CM, Jagura-Burdzy G. 2004. ParB of *Pseudomonas aeruginosa*: interactions with its partner ParA and its target *parS* and specific effects on bacterial growth. *J Bacteriol.* 186:6983–6998.

Casadaban MJ, Cohen SN. Analysis of gene control signals by DNA fusion and cloning in *Escherichia coli*. *J Mol Biol.* 1980 Apr;138(2):179–207. doi: 10.1016/0022-2836(80)90283-1.

Currinn H, Guscott B, Balklava Z, Rothnie A, Wassmer T. APP controls the formation of PI(3,5)P(2) vesicles through its binding of the PIKfyve complex. *Cell Mol Life Sci.* 2016 Jan;73(2):393–408. doi: 10.1007/s00018-015-1993-0.

El-Sayed AK, Hothersall J, Thomas CM. Quorum-sensing dependent regulation of biosynthesis of the polyketide antibiotic mupirocin in *Pseudomonas fluorescens* NCIMB 10586. *Microbiology.* 2001;147:2127–2139.

Glaza P, Osipiuk J, Wenta T, Zurawa-Janicka D, Jarzab M, Lesner A, Banecki B, Skorko-Glonek J, Joachimiak A, Lipinska B. Structural and Functional Analysis of Human HtrA3 Protease and Its Subdomains. *PLoS One.* 2015 Jun 25;10(6):e0131142. doi: 10.1371/journal.pone.0131142.

Holloway BW. Genetic recombination in *Pseudomonas aeruginosa*. *Journal of General Microbiology.* 1995;13:572–581.

Jecz P, Bartosik AA, Glabski K, Jagura-Burdzy G. A single *parS* sequence from the cluster of four sites closest to *oriC* is necessary and sufficient for proper chromosome segregation in *Pseudomonas aeruginosa*. *PLoS One.* 2015 Mar 20;10(3):e0120867. doi: 10.1371/journal.pone.0120867. Erratum in: *PLoS One.* 2016 Mar 24;11(3):e0152541. doi: 10.1371/journal.pone.0152541.

Kawalek A, Glabski K, Bartosik AA, Wozniak D, Kusiak M, Gawor J, Zuchniewicz K, Jagura-Burdzy G. Diverse Partners of the Partitioning ParB Protein in *Pseudomonas aeruginosa*. *Microbiol Spectr.* 2023 Feb 14;11(1):e0428922. doi: 10.1128/spectrum.04289-22.

Kawalek A, Glabski K, Bartosik A.A., Fogtman A., Jagura-Burdzy G. Increased ParB level affects expression of stress response, adaptation and virulence operons and potentiates repression of promoters adjacent to the high affinity binding sites *parS*3 and *parS*4 in *Pseudomonas aeruginosa*. *PLoS ONE.* 2017;12:e0181726.

Kusiak M, Gapczynska A, Plochocka D, Thomas CM, Jagura-Burdzy G. Binding and spreading of ParB on DNA determine its biological function in *Pseudomonas aeruginosa*. *J Bacteriol.* 2011 Jul;193(13):3342–3355. doi: 10.1128/JB.00328-11.

Lasocki K, Bartosik AA, Mierzejewska J, Thomas CM, Jagura-Burdzy G. Deletion of the *parA* (*soj*) homologue in *Pseudomonas aeruginosa* causes ParB instability and affects growth rate, chromosome segregation, and motility. *J Bacteriol.* 2007 Aug;189(15):5762–5772. doi: 10.1128/JB.00371-07.

Lioy VS, Junier I, Lagage V, Vallet I, and Boccard F. Distinct activities of bacterial Condensins for chromosome management in *Pseudomonas aeruginosa*. *Cell reports*. 2020;33:108344. doi.org/10.1016/j.celrep.2020.108344.

Marx CJ, Lidstrom ME. Development of improved versatile broad-host range vectors for use in methylotrophs and other Gram-negative bacteria. *Microbiology.* 2001;147:2065–2075.

Simon R, Priefer U, Puhler A. A broad host mobilization system for *in vivo* genetic engineering: transposon mutagenesis in Gram-negative bacteria. *Bio/Technology.* 1983;1:37–45.

Walter BM, Szulc A, Glinkowska, M. K. Reliable method for high quality His-tagged and untagged *E. coli* phosphoribosyl phosphate synthase (Prs) purification. *Protein Expr. Purif*. 2020, 169. doi:10.1016/j.pep.2020.10558.

Grudniak AM, Klecha B, Wolska KI. Effects of null mutation of the heat-shock gene *htpG* on the production of virulence factors by *Pseudomonas aeruginosa*. *Future Microbiology*. 2018;13:69-80. doi:10.2217/fmb-2017-0111.
